# Differential associations between novel triglyceride-glucose-obesity indices and incident stroke in individuals with early-stage cardiovascular-kidney-metabolic syndrome: a longitudinal cohort study

**DOI:** 10.1186/s12933-026-03240-x

**Published:** 2026-06-08

**Authors:** Zhi-Qing Fu, Li An, Wei Zhang, Yue Lyu, Shan Li

**Affiliations:** 1 Department of Cardiology, The Second Medical Center & National Clinical Research Center for Geriatric Disease, Chinese PLA General Hospital, Beijing, 100853 China; 2 Department of Respiratory and Critical Care Medicine, The Second Medical Center & National Clinical Research Center for Geriatric Disease, Chinese PLA General Hospital, Beijing, 100853 China; 3 Department of Healthcare, The Second Medical Center & National Clinical Research Center for Geriatric Disease, Chinese PLA General Hospital, Beijing, 100853 China

**Keywords:** Insulin resistance, Anthropometric measures, Stroke, CKM syndrome, Cumulative exposure, Longitudinal change

## Abstract

**Background:**

The triglyceride-glucose (TyG) index combined with advanced anthropometric measures has emerged as a promising tool for cardiovascular risk assessment. However, evidence comparing the predictive utility of various novel TyG-obesity indices for incident stroke in individuals with early-stage cardiovascular-kidney-metabolic (CKM) syndrome remains limited. This study aimed to investigate and compare the associations of six novel TyG-obesity indices (TyG-Chinese visceral adiposity index [CVAI], -body roundness index [BRI], -conicity index [CI], -weight-adjusted waist index [WWI], -a body shape index [ABSI], and -relative fat mass [RFM]) with stroke in individuals with CKM stages 0–3.

**Methods:**

This prospective cohort study included 3400 participants from the China Health and Retirement Longitudinal Study. K-means clustering was used to identify longitudinal change patterns, and the Boruta algorithm ranked feature importance. We used Cox proportional hazards models to evaluate the strength of associations, restricted cubic splines (RCS) to explore dose-response relationships, receiver operating characteristic (ROC) curves to assess discriminative ability, and net reclassification improvement (NRI) and integrated discrimination improvement (IDI) to quantify incremental predictive performance.

**Results:**

During a median follow-up of 8.6 years, 232 incident strokes were documented. Using a multidimensional analytical framework incorporating baseline, cumulative, and longitudinal change-based approaches, six indices were differentially associated with stroke risk, with TyG-CVAI and TyG-BRI showing stronger and more consistent associations. Each standard deviation increase in baseline TyG-CVAI and TyG-BRI corresponded to hazard ratios (HRs) of 1.36 (95% CI 1.17–1.57) and 1.32 (1.14–1.53). The corresponding cumulative HRs were 1.31 (1.14–1.52) and 1.29 (1.11–1.49). Compared with the stable low-level group, sustained high-level group yielded HRs of 2.28 (1.54–3.39) for TyG-CVAI and 2.01 (1.35–2.99) for TyG-BRI. Stage-specific analyses revealed stronger associations in CKM stage 2. Both indices demonstrated positive linear dose-response relationships. ROC analyses showed that TyG-CVAI and TyG-BRI had relatively better discrimination, and NRI/IDI analyses demonstrated incremental predictive value over the baseline model.

**Conclusion:**

Among the six novel TyG-obesity indices evaluated, TyG-CVAI and TyG-BRI emerged as stronger predictors of incident stroke in individuals with early-stage CKM syndrome, particularly in stage 2. These findings highlight the potential utility of these two indices for stroke risk stratification and may inform early detection strategies in this vulnerable population.

**Graphical abstract:**

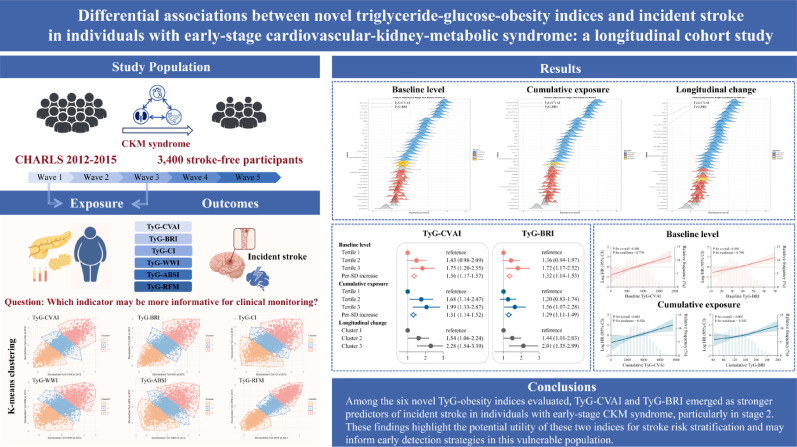

**Supplementary Information:**

The online version contains supplementary material available at 10.1186/s12933-026-03240-x.

## Research insights

**What is already known?**

- Integrating insulin resistance and obesity assessments improves cardiovascular risk prediction.
- The TyG index combined with traditional obesity indices (BMI, WC, WHtR) is a validated, cost-effective risk marker.
- Novel anthropometric measures more accurately capture visceral fat accumulation and demonstrate stronger associations with cardiovascular risk than traditional indices.

**What is the key research question?**

- Can the TyG index combined with novel obesity indices predict stroke risk in early-stage CKM syndrome? Which of the six novel indices (TyG-CVAI, -BRI, -CI, -WWI, -ABSI, -RFM) demonstrate better predictive performance? Does the predictive utility vary across CKM stages?

**What is new?**

- Baseline measurements and longitudinal exposure of novel TyG-obesity indices were differentially associated with incident stroke in early-stage CKM syndrome.
- TyG-CVAI and TyG-BRI showed stronger associations with stroke across multidimensional assessments, including baseline, cumulative, and longitudinal change based analyses.
- Their associations were more pronounced in CKM stage 2, the largest subgroup accounting for more than half of the cohort, suggesting their potential as risk markers in this asymptomatic yet metabolically vulnerable population.

**What are the clinical implications?**

- Although novel TyG-obesity indices show potential for stroke prediction in CKM syndrome, their associations with incident stroke vary across different indices.
- Whether applied as static baseline measures or dynamic longitudinal monitoring tools, TyG-CVAI and TyG-BRI could contribute to early stroke risk stratification.
- These indices could inform personalized prevention strategies, particularly in CKM stage 2, which represents a window for closer surveillance and earlier intervention.

## Introduction

According to the Global Burden of Disease 2021 report, stroke accounts for approximately 7.3 million deaths annually, ranking as the third leading cause of mortality worldwide after ischemic heart disease and COVID-19 [1]. Beyond its high mortality, stroke often causes persistent functional impairment, imposing sustained strain on families and healthcare systems. In China, the age-standardized incidence of stroke surged by 86% between 1990 and 2019, making it the predominant contributor to mortality and disability [2]. Given this escalating burden, the identification of cost-effective and reliable indicators for early risk stratification has become an important public health consideration.

In 2023, the American Heart Association (AHA) introduced the cardiovascular-kidney- metabolic (CKM) syndrome conceptual framework, highlighting the interconnected pathophysiology of cardiovascular, kidney, and metabolic diseases [3]. CKM syndrome is highly prevalent: over 90% of U.S. adults meet the criteria for stage 1 or higher, and approximately 15% progress to stage 4, a pattern that substantially increases the risk of premature mortality [4]. Stages 0–3 represent the asymptomatic preclinical phases, during which preventive strategies may offer the greatest benefit. As stroke is a major clinical concern in advanced CKM syndrome, identifying reliable predictors during the early, modifiable stages (0–3) is clinical important [3].

Insulin resistance (IR) and obesity are central to the pathogenesis of CKM syndrome [5, 6]. The triglyceride-glucose (TyG) index serves as a cost-effective proxy for IR due to its simplicity and accessibility [7]. Elevated TyG levels have been linked to a wide range of of adverse outcomes, including atherosclerotic cardiovascular disease, decompensated heart failure, acute renal impairment, surgical critical illness, cardiac arrest, and mortality [8–13]. To capture the synergistic effects of IR and adiposity, the TyG index has been combined with traditional anthropometric measures, yielding composite indices such as TyG-body mass index (BMI), TyG-waist circumference (WC), and TyG-waist-to-height ratio (WHtR) [14–16]. Accumulating evidence indicates that these composite indices outperform the TyG index alone in predicting cardiometabolic risk [17–19].

While most previous studies have focused on combining the TyG index with traditional obesity indices, the integration of TyG with novel obesity metrics, including the Chinese Visceral Adiposity Index (CVAI), Body Roundness Index (BRI), Conicity Index (CI), Weight-Adjusted Waist Index (WWI), A Body Shape Index (ABSI), and Relative Fat Mass (RFM), remains largely unexplored. Existing evidence is confined to single or a limited number of indices, with considerable heterogeneity in study populations, outcome definitions, and analytical approaches. Critically, no study has systematically compared TyG index combined with these advanced anthropometric measures within a CKM cohort to determine their relative predictive value for stroke risk. Furthermore, it remains unknown whether the strength of associations varies across CKM stages, a question with potential implications for more refined, stage-specific stratification.

This study systematically evaluate the associations between six novel TyG-derived indices (TyG-CVAI, TyG-BRI, TyG-CI, TyG-WWI, TyG-ABSI, and TyG-RFM) and incident stroke in individuals with CKM stages 0–3. Using a multidimensional framework integrating baseline, cumulative, and longitudinal change analyses, we compare their predictive performance and explore stage-specific relationships. The findings may help identify indicators that are more informative for risk stratification and provide insights for early prevention strategies.

## Methods

### Study population

The China Health and Retirement Longitudinal Study (CHARLS) is an ongoing national cohort study designed to evaluate the health and socioeconomic status of community-dwelling adults [20]. The initial survey was conducted in 2011–2012 (wave 1), with subsequent surveys taking place in 2013 (wave 2), 2015 (wave 3), 2018 (wave 4), and 2020 (wave 5). The study protocol was approved by the Ethical Review Committee of Peking University (IRB: 00001052–11015), and all participants provided written informed consent. The study adhered to the Declaration of Helsinki.

**Fig. 1:**
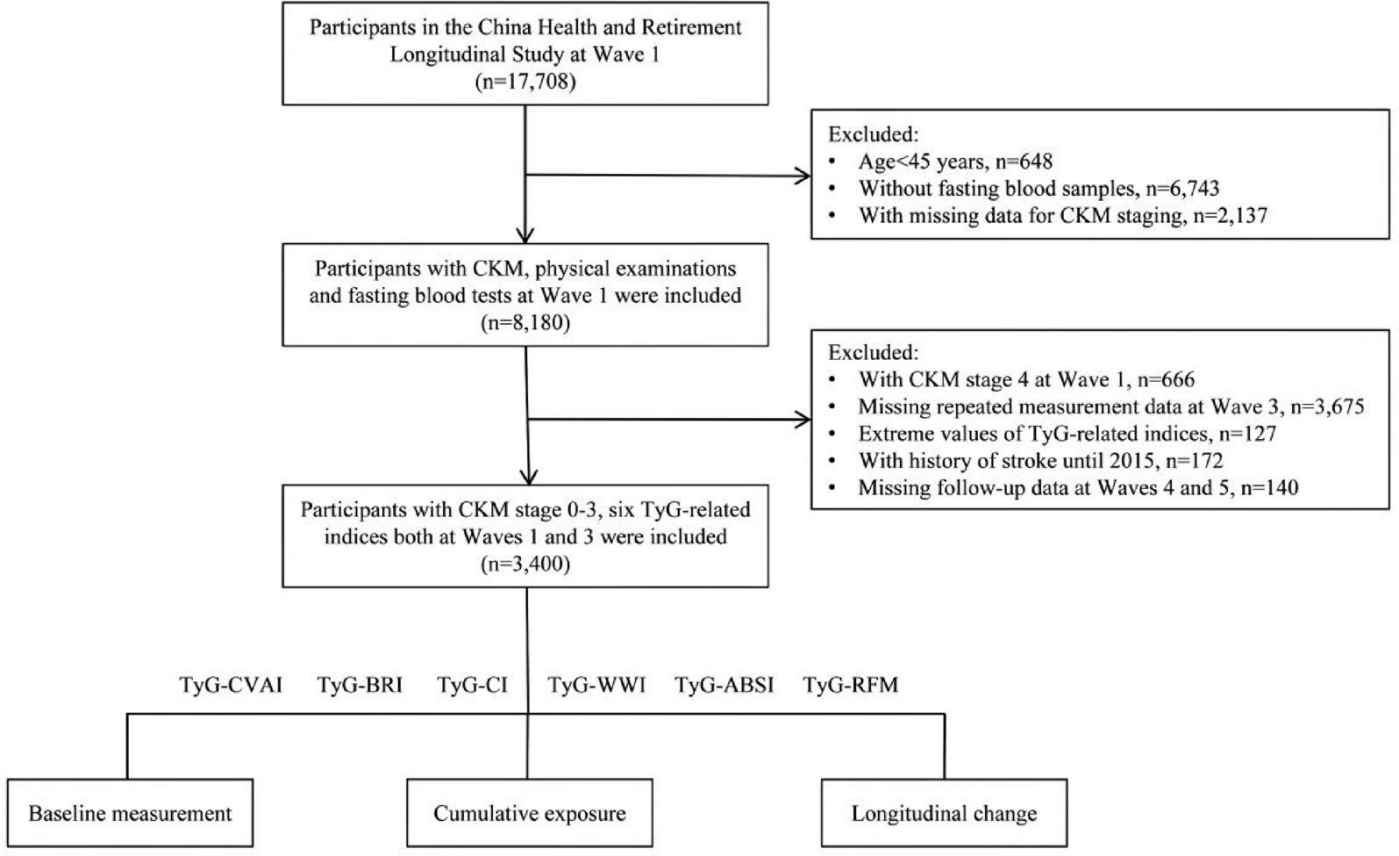
Flow chart for eligible participants

Of the 17,708 participants enrolled at wave 1, 9,528 were excluded because of age < 45 years, missing fasting blood measurements, or incomplete CKM staging data. The remaining 8,180 participants underwent further exclusion: CKM stage 4, missing wave 3 repeated measurements, extreme TyG-related index values, stroke between waves 1 and 3, or missing follow-up data at waves 4 or 5. After these exclusions, 3,400 participants were included in the final analysis. Baseline assessments used wave 1 data, whereas cumulative exposure and longitudinal change analyses used repeated measurements from waves 1 and 3 (Fig. 1).

### Data collection

Standardized questionnaires and examinations were used to collect multidimensional data, which included: [1] demographic and lifestyle characteristics (age, sex, educational attainment, urban/rural residence, marital status, smoking, and alcohol consumption); [2] physical examinations (weight, height, waist circumference, systolic blood pressure [SBP], and diastolic blood pressure [DBP]); [3] laboratory parameters (fasting plasma glucose (FPG), total cholesterol, triglycerides, low-density lipoprotein cholesterol [LDL-C], high-density lipoprotein cholesterol [HDL-C], glycated hemoglobin [HbA1c], creatinine, uric acid, and hemoglobin); [4] medical history (hypertension, diabetes, chronic kidney disease [CKD], and chronic lung disease); and [5] medication use (antidiabetic, antihyperlipidemic, and antihypertensive agents). Educational level was categorized as high school or above versus below high school. Hukou status (household registration) was classified as rural or non-rural. Marital status was defined as married versus other (separated, widowed, divorced, never married). Smoking status was dichotomized as current smokers (≥ 100 lifetime cigarettes and not quit in the past year) versus non-current smokers. Alcohol intake was similarly dichotomized as current drinkers (≥ once per month and not quit in the past year) versus non-current drinkers. Estimated glomerular filtration rate (eGFR) was calculated using the 2009 CKD- EPI equation. Diagnostic criteria for underlying medical conditions are provided in Table S1.

### Definitions of CKM syndrome stages 0–3

CKM syndrome was staged at baseline (wave 1) according to the AHA criteria [3]. Stage 0: no identifiable CKM risk factors. Stage 1: overweight, visceral obesity, or dysfunctional adipose tissue, without CKD. Stage 2: presence of metabolic risk factors (e.g., dyslipidemia, hypertension, metabolic syndrome, or type 2 diabetes) and/or CKD. Stage 3: subclinical cardiovascular disease (CVD) or risk equivalents (Table S2). Because the CHARLS dataset lacked direct imaging or biomarker evidence of subclinical CVD (e.g., coronary angiography, CT angiography, or echocardiography), we operationally defined stage 3 using high-risk equivalents: an estimated 10-year CVD risk ≥ 20% based on the PREVENT equations [21], or very high-risk CKD at stages G4 or G5 [22] (Table S3).

### Definitions of novel TyG-obesity indices

Six advanced anthropometric metrics (CVAI, BRI, CI, WWI, ABSI and RFM) have been previously defined and validated [23, 24]. We constructed the corresponding TyG-obesity indices by multiplying the TyG index by each metric (e.g., TyG-CVAI = TyG×CVAI). To quantify cumulative exposure, we referred to the concept of concentration-time integration. Taking TyG-CVAI as an example, cumulative TyG-CVAI = (TyG-CVAI_2012_ + TyG-CVAI_2015_)/2 × ΔTime, where ΔTime is the time interval between the two measurements in 2012 and 2015. Detailed calculation formulas and values are provided in Tables S4 and S5.

**Fig. 2:**
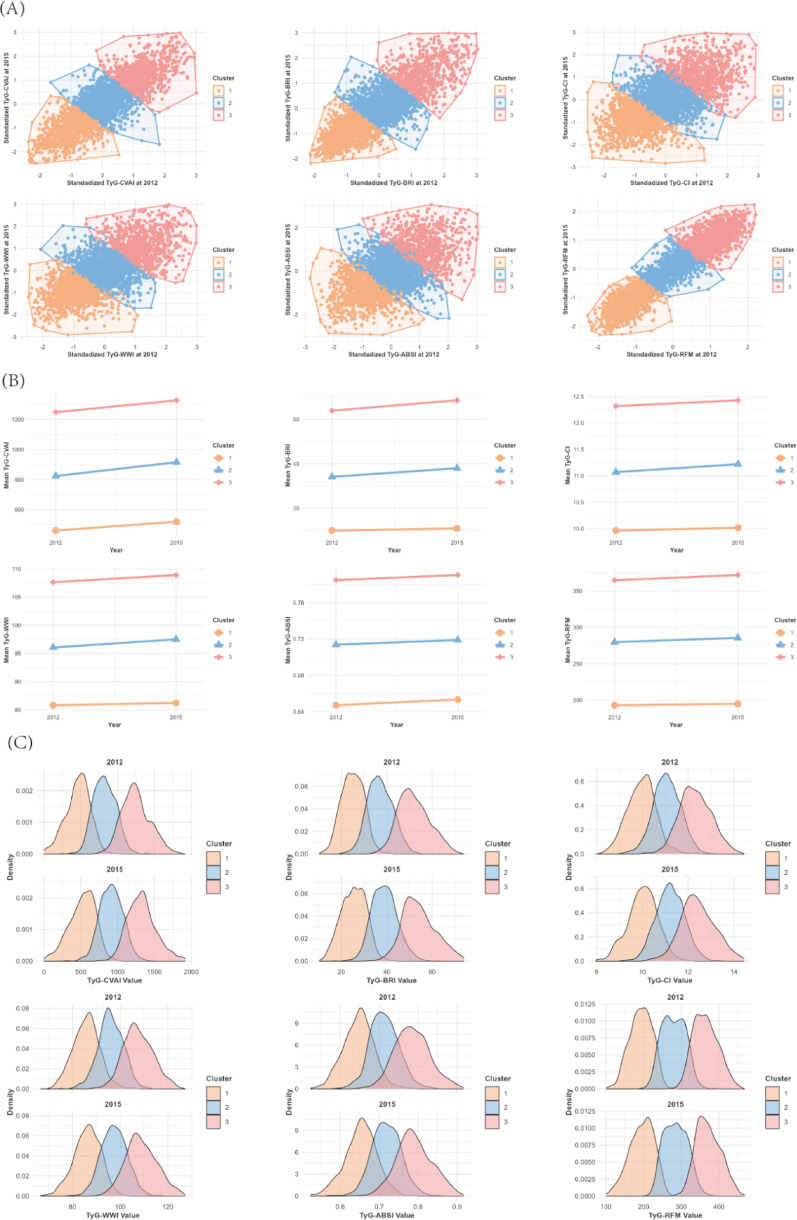
Clustering of longitudinal changes in TyG-obesity indices from 2012 to 2015. **A** Scatter plots showing standardized TyG-obesity indices in 2012 (x-axis) and 2015 (y-axis), colored according to three distinct clusters derived from K-means clustering (Euclidean distance). **B** Line plots showing the mean TyG-obesity index changes for the three clusters over the period 2012–2015. **C** Density plots showing the distributions of TyG-obesity indices within each cluster in 2012 and 2015

K-means clustering was used to classify participants into three clinically meaningful groups based on longitudinal changes in TyG-obesity indices measured in 2012 and 2015. Cluster 1 (persistently low level): indices remained stable at low levels, indicating sustained metabolic control. Cluster 2 (moderate level): indices remained at intermediate levels, suggesting moderately controlled metabolic status. Cluster 3 (persistently high level): indices were high at baseline and increased further, reflecting progressive metabolic dysregulation (Fig. 2).

### Study endpoints

The primary endpoint was incident stroke. Stroke events occurring during waves 4 and 5 were captured to maximize ascertainment and reduce underreporting. Events were primarily identified through self-reported data from computer-assisted standardized questionnaires, with rigorous quality control measures implemented by the CHARLS team to ensure reliability.

### Statistical analysis

Statistical analyses were performed using R software (version 4.3.2). Continuous variables were expressed as mean ± standard deviation (SD) or median (interquartile range), and categorical variables as frequencies (percentages). Group comparisons were conducted using the chi-square test, Kruskal-Wallis test, or Mann-Whitney U test. K-means clustering (Euclidean distance) was used to identify longitudinal change patterns. This unsupervised method groups individuals based on baseline values and changes between two time points, capturing subtle variations in dynamic patterns [25]. All indices were standardized (z-score) before clustering to ensure comparability. The optimal number of clusters was determined by the elbow method, and the within-cluster sum of squares leveled off at k = 3, indicating that three clusters balanced model parsimony and explanatory accuracy while avoiding overfitting (Fig. S1). Each participant was assigned to the cluster with the nearest centroid. The Boruta algorithm, a random forest-based feature selection method, was used to rank variable/feature importance. It compares original features with randomly generated shadow features, using 500 trees for stable estimates and 100 iterations until all features are confirmed or rejected (Fig. S2). This procedure was applied both to evaluate the relative performance of the TyG-obesity indices themselves and to rank covariates for subsequent model adjustment.

Cox proportional hazards models were used to assess the associations of each TyG-obesity index with incident stroke across three analytical frameworks: baseline level, cumulative exposure, and longitudinal change pattern. Model 1 was unadjusted. Model 2 adjusted for age and sex. Model 3 further adjusted for smoking, alcohol use, SBP, DBP, marital status, household registration, educational attainment, HDL-C, LDL-C, uric acid, hemoglobin, eGFR, medical history, and medication use. Covariates were selected based on Boruta algorithm rankings, and meanwhile taking the clinical relevance and prior evidence into consideration [26]. The proportional hazards assumption was verified using Schoenfeld residuals, and no violations were observed (Supplementary material 2: Table S1). To avoid multicollinearity, covariates with a variance inflation factor ≥ 5 were removed (Supplementary material 2: Table S2). Most of the covariates were complete, and missing data for hemoglobin (*n* = 29, 0.85%) and hukou status (*n* = 3, 0.09%) were handled by multiple imputation using chained equations. To examine whether the association varied by CKM stage, we conducted stage-specific analyses by stratifying participants into CKM stages 0–1, 2, and 3. Dose-response relationships were evaluated using restricted cubic splines (RCS) with three knots placed at the 10th, 50th, and 90th percentiles of each index, and linearity was tested by the likelihood-ratio test.

Receiver operating characteristic (ROC) curves were used to assess predictive performance, and area under the curve (AUC) values were compared using DeLong’s test. To further quantify the incremental predictive value of the optimal TyG-obesity indices beyond the basic model comprising conventional risk factors, we calculated the continuous net reclassification improvement (NRI) and integrated discrimination improvement (IDI), along with 95% CIs. Decision curve analysis (DCA) was performed to evaluate clinical utility by estimating the net benefit of incorporating the optimal TyG-obesity indices into the basic model across a range of threshold probabilities for stroke. Mediation analysis was applied to explore whether estimated pulse wave velocity (ePWV), a measure of arterial stiffness, mediated the association between TyG-derived indices and stroke.

Sensitivity analyses were conducted to assess the robustness of the findings. First, to address reverse causality, we repeated the primary analysis after excluding participants diagnosed with stroke at wave 4. Second, stratified analyses by age, sex, smoking, alcohol use, hypertension, and diabetes were performed to explore potential effect modification. The Benjamini-Hochberg false discovery rate (FDR) procedure was applied to control for multiplicity in subgroup and interaction tests, with the corresponding q-values being reported. Third, we applied the Fine-Gray competing risks model to account for all-cause mortality as a competing event.

## Results

Of the 3400 participants included in the analysis, 1,564 (46.0%) were male, with a mean age of 58.3 ± 8.2 years. During a median follow-up of 8.6 years, 232 incident strokes (6.8%) occurred. Compared with those who remained stroke-free, participants who developed stroke were older and had less favorable cardiometabolic and sociodemographic profiles at baseline. They had higher body weight and waist circumference, elevated blood pressure, and were more likely to reside in rural areas and to have lower educational attainment. Biochemically, the stroke group showed higher levels of FPG, triglycerides, LDL-C, HbA1c, and uric acid, whereas HDL-C and eGFR were lower. The prevalence of hypertension was higher in the stroke group, and a larger proportion was classified as CKM stage 2 or higher, with fewer classified as stages 0–1. Notably, both baseline levels and cumulative exposure of the six indices were significantly higher among those who later experienced stroke. Regarding longitudinal change patterns, participants who subsequently developed stroke were more frequently assigned to the high-risk metabolic cluster (cluster 3) and less frequently to the low-risk cluster (cluster 1) (Table 1).

**Table 1:** Baseline characteristics of participants

Variables	Total (*n* = 3400)	Non-stroke (*n* = 3168)	Stroke (*n* = 232)	*P* value
Age, years	58.3 ± 8.2	58.2 ± 8.3	60.4 ± 7.9	< 0.001
Male, n (%)	1564 (46.0)	1458 (46.0)	106 (45.7)	0.922
Current smokers, n (%)	1035 (30.4)	965 (30.5)	70 (30.2)	0.927
Current drinkers, n (%)	1152 (33.9)	1075 (33.9)	77 (33.2)	0.817
Systolic blood pressure, mmHg	127.5 ± 20.1	127.1 ± 20.0	132.9 ± 21.9	< 0.001
Diastolic blood pressure, mmHg	74.7 ± 11.8	74.5 ± 11.7	76.8 ± 12.8	0.004
Married status, n (%)	3061 (90.0)	2864 (90.4)	197 (84.9)	0.007
Rural Hukou status	2905 (85.5)	2698 (85.2)	207 (89.6)	0.067
High school education or above, n (%)	305 ( 9.0)	289 (9.1)	16 (6.9)	0.252
Fasting plasma glucose, mg/dL	105.6 ± 22.4	105.4 ± 22.4	107.7 ± 23.1	0.139
Total cholesterol, mg/dL	193.1 ± 37.0	192.5 ± 36.9	201.2 ± 37.7	< 0.001
Triglycerides, mg/dL	100.0 (72.6, 142.5)	100.0 (71.7, 141.6)	105.3 (83.2, 154.0)	< 0.001
HDL-C, mg/dL	51.9 ± 14.7	52.2 ± 14.6	48.7 ± 14.9	< 0.001
LDL-C, mg/dL	118.2 ± 33.2	117.6 ± 33.1	126.7 ± 34.5	< 0.001
HbA1c, %	5.2 ± 0.6	5.2 ± 0.6	5.3 ± 0.7	0.031
Uric acid, mg/dL	4.3 ± 1.2	4.3 ± 1.2	4.5 ± 1.3	0.008
Hemoglobin, g/dL	14.4 ± 2.2	14.4 ± 2.2	14.7 ± 2.4	0.088
eGFR, mL/min/1.73m^2^	97.4 ± 12.6	97.6 ± 12.5	94.8 ± 12.7	< 0.001
CKM syndrome, n (%)				< 0.001
Stage 0	417 (12.3)	406 (12.8)	11 (4.7)	
Stage 1	913 (26.9)	866 (27.3)	47 (20.3)	
Stage 2	1744 (51.3)	1592 (50.3)	152 (65.5)	
Stage 3	326 ( 9.6)	304 (9.6)	22 (9.5)	
Hypertension, n (%)	1255 (36.9)	1136 (35.9)	119 (51.3)	< 0.001
Diabetes mellitus, n (%)	414 (12.2)	382 (12.1)	32 (13.8)	0.435
Chronic kidney disease, n (%)	222 ( 6.5)	204 (6.4)	18 (7.8)	0.432
Lung disease, n (%)	276 (8.1)	251 (7.9)	25 (10.8)	0.125
Antihypertensive agents, n (%)	519.0 (15.3)	461.0 (14.5)	58.0 (25.0)	< 0.001
Antidiabetic agents, n (%)	90.0 ( 2.6)	78.0 (2.46	12.0 (5.1)	0.042
Antihyperlipidemic agents, n (%)	138.0 ( 4.1)	115.0 (3.6)	23.0 (9.9)	< 0.001
Baseline TyG-CVAI	805.3 ± 342.9	794.7 ± 339.7	950.4 ± 353.1	< 0.001
Baseline TyG-BRI	35.8 ± 11.7	35.5 ± 11.5	40.3 ± 12.8	< 0.001
Baseline TyG-CI	11.0 ± 1.1	11.0 ± 1.1	11.4 ± 1.1	< 0.001
Baseline TyG-WWI	95.7 ± 10.0	95.4 ± 9.9	99.1 ± 9.8	< 0.001
Baseline TyG-ABSI	0.71 ± 0.07	0.71 ± 0.07	0.73 ± 0.06	< 0.001
Baseline TyG-RFM	282.4 ± 78.7	281.2 ± 78.5	298.5 ± 79.2	0.001
Cumulative TyG-CVAI	3368.4 ± 1333.5	3328.6 ± 1321.7	3912.5 ± 1377.3	< 0.001
Cumulative TyG-BRI	146.0 ± 46.1	144.8 ± 45.4	162.7 ± 51.3	< 0.001
Cumulative TyG-CI	44.2 ± 4.1	44.1 ± 4.1	45.5 ± 4.1	< 0.001
Cumulative TyG-WWI	383.8 ± 37.9	382.9 ± 37.7	395.3 ± 38.6	< 0.001
Cumulative TyG-ABSI	2.85 ± 0.24	2.85 ± 0.24	2.92 ± 0.24	< 0.001
Cumulative TyG-RFM	1136.6 ± 314.0	1132.4 ± 313.3	1193.8 ± 318.3	0.004
TyG-CVAI longitudinal change pattern, n (%)				< 0.001
Cluster 1	1221 (35.9)	1173 (37.0)	48 (20.7)	
Cluster 2	1434 (42.2)	1339 (42.3)	95 (40.9)	
Cluster 3	745 (21.9)	656 (20.7)	89 (38.4)	
TyG-BRI longitudinal change pattern, n (%)				< 0.001
Cluster 1	1293 (38.0)	1235 (39.0)	58 (25.0)	
Cluster 2	1394 (41.0)	1299 (41.0)	95 (40.9)	
Cluster 3	713 (21.0)	634 (20.0)	79 (34.1)	
TyG-CI longitudinal change pattern, n (%)				< 0.001
Cluster 1	1193 (35.1)	1148 (36.2)	45 (19.4)	
Cluster 2	1507 (44.3)	1388 (43.8)	119 (51.3)	
Cluster 3	700 (20.6)	632 (19.9)	68 (29.3)	
TyG-WWI longitudinal change pattern, n (%)				< 0.001
Cluster 1	1115 (32.8)	1064 (33.6)	51 (22)	
Cluster 2	1495 (44.0)	1389 (43.8)	106 (45.7)	
Cluster 3	790 (23.2)	715 (22.6)	75 (32.3)	
TyG-ABSI longitudinal change pattern, n (%)				< 0.001
Cluster 1	1166 (34.3)	1109 (35.0)	57 (24.6)	
Cluster 2	1486 (43.7)	1383 (43.7)	103 (44.4)	
Cluster 3	748 (22.0)	676 (21.3)	72 (31.0)	
TyG-RFM longitudinal change pattern, n (%)				0.010
Cluster 1	1232 (36.2)	1158 (36.6)	74 (31.9)	
Cluster 2	1080 (31.8)	1017 (32.1)	63 (27.2)	
Cluster 3	1088 (32.0)	993 (31.3)	95 (40.9)	

CKM syndrome, cardiovascular kidney metabolic syndrome. HbA1c, glycated haemoglobin. HDL-C, high-density lipoprotein cholesterol. LDL-C, low-density lipoprotein cholesterol. TyG, triglyceride-glucose index. CVAI, Chinese visceral adiposity index. BRI, body roundness index. CI, conicity index. ABSI, a body shape index. WWI, weight-adjusted waist index. RFM, relative fat mass

### Feature importance

**Fig. 3:**
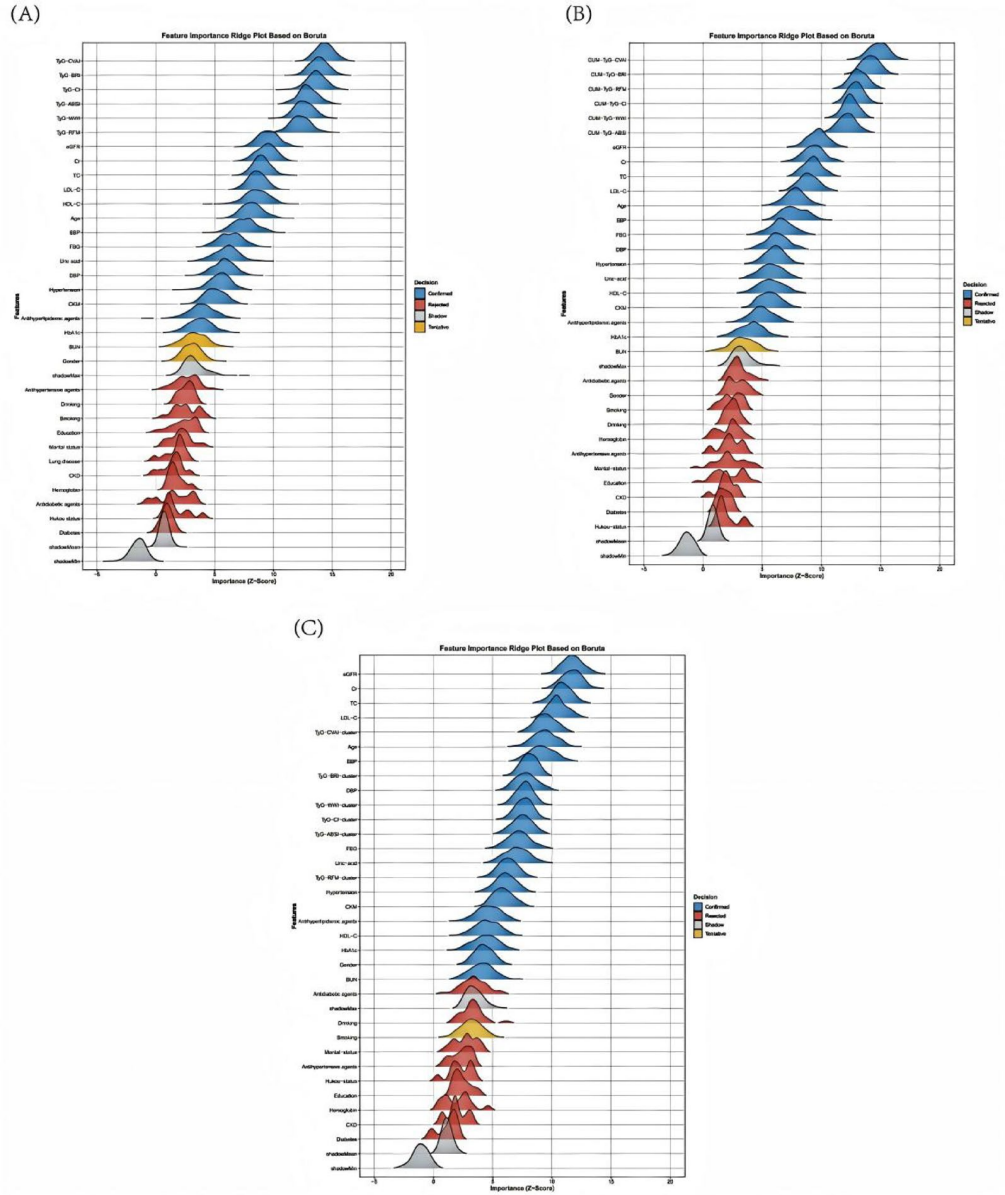
Boruta feature importance ridge plot for stroke in CKM syndrome stages 0–3. **A** Baseline TyG-obesity indices. **B** Cumulative TyG-obesity indices. **C** Longitudinal change of TyG-obesity indices. Blue areas indicate confirmed features, red areas indicate rejected features, yellow areas indicate tentative features, and grey areas indicate shadow features.

The Boruta algorithm was applied to rank feature importance across three analytical dimensions (baseline, cumulative, and longitudinal change). All six TyG-obesity indices were consistently identified as significant features. Notably, the baseline and cumulative values of TyG-CVAI and TyG-BRI ranked among the top two features overall. In the longitudinal change analysis, although certain renal and lipid parameters exhibited higher relative importance (Z-scores), TyG-CVAI and TyG-BRI remained the most prominent among the six TyG-derived indices, underscoring their primacy within this category. Other important features included age, sex, systolic and diastolic blood pressure, eGFR, LDL-C, HDL-C and uric acid, all of which were retained as covariates for the subsequent model adjustment. Although variables such as smoking, alcohol consumption, diabetes, and CKD showed lower relative importance (Z-scores), they were included in the model adjustment based on clinical relevance and prior evidence (Fig. 3).

### Associations between novel TyG-obesity indices and stroke

All baseline indices except TyG-RFM showed a graded increase in stroke risk across tertiles. In the fully adjusted models, the hazard ratios (HRs) for the highest versus lowest tertile were: TyG-CVAI, 1.75 (95% CI 1.20–2.55); TyG-BRI, 1.72 (1.17–2.52); TyG-CI, 1.92 (1.32–2.80); TyG-WWI, 1.81 (1.23–2.66); and TyG-ABSI, 1.74 (1.20–2.50). After Z-score standardization, each SD increase in baseline levels was associated with a 36%, 32%, 29%, 28%, and 24% higher risk of stroke, respectively. Analyses treating the indices as both continuous and categorical variables indicated that baseline TyG-CVAI and TyG-BRI had stronger associations with stroke onset (Table 2).

**Table 2:** Association between baseline TyG-obesity indices and stroke in CKM syndrome stages 0–3

Variables	Event (%)	Model I		Model II		Model III	
		HR (95% CI)	*P* value	HR (95% CI)	*P* value	HR (95% CI)	*P* value
*Baseline TyG-CVAI*							
Tertile 1 (2.78-631.39)	45 (4.0)	reference		reference		reference	
Tertile 2 (631.42-937.91)	77 (6.8)	1.67 (1.15 ~ 2.41)		1.59 (1.10 ~ 2.31)		1.43 (0.98 ~ 2.09)	
Tertile 3 (938.09-1895.71)	110 (9.7)	2.44 (1.72 ~ 3.45)	< 0.001*	2.24 (1.58 ~ 3.19)	< 0.001*	1.75 (1.20 ~ 2.55)	0.004*
*BaselineTyG-BRI*							
Tertile 1 (11.27–29.54)	50 (4.4)	reference		reference		reference	
Tertile 2 (29.55–39.99)	74 (6.5)	1.45 (1.01 ~ 2.07)		1.53 (1.07 ~ 2.21)		1.36 (0.94 ~ 1.97)	
Tertile 3 (40.01–73.01)	108 (9.5)	2.10 (1.50 ~ 2.93)	< 0.001*	2.20 (1.55 ~ 3.12)	< 0.001*	1.72 (1.17 ~ 2.52)	0.006*
*Baseline TyG-CI*							
Tertile 1 (8.27–10.49)	44 (3.9)	reference		reference		reference	
Tertile 2 (10.49–11.46)	81 (7.1)	1.84 (1.27 ~ 2.65)		1.77 (1.22 ~ 2.56)		1.65 (1.13 ~ 2.39)	
Tertile 3 (11.47–14.40)	107 (9.4)	2.50 (1.76 ~ 3.55)	< 0.001*	2.35 (1.64 ~ 3.36)	< 0.001*	1.92 (1.32 ~ 2.80)	0.001*
*Baseline TyG-WWI*							
Tertile 1 (71.27–90.88)	47 (4.1)	reference		reference		reference	
Tertile 2 (90.89–99.81)	77 (6.8)	1.65 (1.15 ~ 2.38)		1.65 (1.14 ~ 2.38)		1.49 (1.03 ~ 2.16)	
Tertile 3 (99.82-128.52)	108 (9.5)	2.32 (1.65 ~ 3.27)	< 0.001*	2.26 (1.57 ~ 3.25)	< 0.001*	1.81 (1.23 ~ 2.66)	0.004*
*Baseline TyG-ABSI*							
Tertile 1 (0.53–0.67)	47 (4.1)	reference		reference		reference	
Tertile 2 (0.68–0.73)	82 (7.2)	1.80 (1.26 ~ 2.58)		1.70 (1.18 ~ 2.44)		1.57 (1.09 ~ 2.26)	
Tertile 3 (0.74–0.93)	103 (9.1)	2.30 (1.63 ~ 3.25)	< 0.001*	2.07 (1.46 ~ 2.96)	< 0.001*	1.74 (1.20 ~ 2.50)	0.005*
*Baseline TyG-RFM*							
Tertile 1 (102.91-235.15)	68 (6.0)	reference		reference		reference	
Tertile 2 (235.21-329.06)	70 (6.2)	0.96 (0.69 ~ 1.35)		1.31 (0.85 ~ 1.92)		1.15 (0.76 ~ 1.74)	
Tertile 3 (329.21-456.64)	94 (8.3)	1.30 (0.95 ~ 1.78)	0.081*	1.78 (0.98 ~ 2.85)	0.070*	1.58 (0.87 ~ 2.56)	0.124*
*Per SD increase*							
Baseline TyG-CVAI	232 (6.8)	1.52 (1.34 ~ 1.72)	< 0.001	1.46 (1.28 ~ 1.66)	< 0.001	1.36 (1.17 ~ 1.57)	< 0.001
Baseline TyG-BRI	232 (6.8)	1.41 (1.25 ~ 1.59)	< 0.001	1.45 (1.27 ~ 1.65)	< 0.001	1.32 (1.14 ~ 1.53)	< 0.001
Baseline TyG-CI	232 (6.8)	1.44 (1.27 ~ 1.63)	< 0.001	1.40 (1.23 ~ 1.59)	< 0.001	1.29 (1.12 ~ 1.49)	< 0.001
Baseline TyG-WWI	232 (6.8)	1.41 (1.24 ~ 1.60)	< 0.001	1.39 (1.22 ~ 1.60)	< 0.001	1.28 (1.10 ~ 1.48)	0.001
Baseline TyG-ABSI	232 (6.8)	1.38 (1.22 ~ 1.57)	< 0.001	1.32 (1.16 ~ 1.50)	< 0.001	1.24 (1.08 ~ 1.42)	0.003
Baseline TyG-RFM	232 (6.8)	1.21 (1.06 ~ 1.38)	0.005	1.68 (1.28 ~ 2.39)	< 0.001	1.59 (1.21 ~ 2.09)	0.001

Model I unadjusted. Model II adjusted for age and sex. Model III adjusted for age, sex, smoking, drinking, SBP, DBP, marital status, hukou status, education, HDL-C, LDL-C, uric acid, hemoglobin, eGFR, hypertension, diabetes, CKD, chronic lung disease, antihypertensive agents, antidiabetic agents and antihyperlipidemic agents.*P for trend.

A similar risk gradient was observed for cumulative exposure between 2012 and 2015. The highest tertiles of cumulative TyG-CVAI (HR 1.99, 95% CI 1.33–2.87), TyG-BRI (1.56, 1.07–2.28), TyG-WWI (1.58, 1.08–2.26), and TyG-ABSI (1.42, 1.00-2.02) were associated with increased stroke risk, whereas TyG-CI and TyG-RFM showed no significant trends. For each SD increase in cumulative exposure, the stroke risk increased by 31% for TyG-CVAI and 29% for TyG-BRI, whereas the risk increases for cumulative TyG-WWI and TyG-ABSI were not statistically significant (Table 3).

**Table 3:** Association between cumulative exposure and longitudinal change pattern of TyG-obesity indices and stroke in CKM syndrome stages 0–3

Variables	Event (%)	Model I		Model II		Model III	
		HR (95% CI)	*P* value	HR (95% CI)	*P* value	HR (95% CI)	*P* value
*Cumulative TyG-CVAI*							
Tertile 1 (87.65-2689.79)	42 (3.7)	reference		reference		reference	
Tertile 2 (2689.95-3925.96)	79 (7.0)	1.85 (1.27 ~ 2.69)		1.84 (1.26 ~ 2.69)		1.68 (1.14 ~ 2.47)	
Tertile 3 (3926.22-7602.28)	111 (9.8)	2.59 (1.82 ~ 3.70)	< 0.001*	2.43 (1.70 ~ 3.48)	< 0.001*	1.99 (1.33 ~ 2.87)	0.001*
*Cumulative TyG-BRI*							
Tertile 1 (43.70-120.43)	54 (4.8)	reference		reference		reference	
Tertile 2 (120.51-162.14)	70 (6.2)	1.27 (0.89 ~ 1.81)		1.37 (0.95 ~ 1.96)		1.20 (0.83 ~ 1.74)	
Tertile 3 (162.29-292.28)	108 (9.5)	1.89 (1.36 ~ 2.62)	< 0.001*	2.02 (1.43 ~ 2.86)	< 0.001*	1.56 (1.07 ~ 2.28)	0.018*
*Cumulative TyG-CI*							
Tertile 1 (38.25–41.94)	43 (3.8)	reference		reference		reference	
Tertile 2 (42.95–45.84)	84 (7.4)	1.53 (1.08 ~ 2.18)		1.52 (1.06 ~ 2.16)		1.37 (0.95 ~ 1.96)	
Tertile 3 (46.92–56.88)	105 (9.3)	1.93 (1.38 ~ 2.71)	< 0.001*	1.84 (1.30 ~ 2.60)	< 0.001*	1.44 (1.00 ~ 2.07)	0.064*
*Cumulative TyG-WWI*							
Tertile 1 (282.51-365.17)	52 (4.6)	reference		reference		reference	
Tertile 2 (365.26-399.16)	71 (6.3)	1.29 (0.91 ~ 1.85)		1.34 (0.93 ~ 1.92)		1.21 (0.84 ~ 1.74)	
Tertile 3 (399.17-505.17)	109 (9.6)	2.00 (1.43 ~ 2.78)	< 0.001*	2.00 (1.40 ~ 2.86)	< 0.001*	1.58 (1.08 ~ 2.26)	0.015*
*Cumulative TyG-ABSI*							
Tertile 1 (2.17–2.73)	54 (4.8)	reference		reference		reference	
Tertile 2 (2.74–2.95)	71 (6.3)	1.24 (0.87 ~ 1.76)		1.20 (0.84 ~ 1.71)		1.13 (0.79 ~ 1.62)	
Tertile 3 (2.95–3.61)	107 (9.4)	1.91 (1.37 ~ 2.64)	< 0.001*	1.74 (1.25 ~ 2.45)	0.001*	1.42 (1.00 ~ 2.02)	0.044*
*Cumulative TyG-RFM*							
Tertile 1 (686.61-940.83)	67 (5.9)	reference		reference		reference	
Tertile 2 (1034.83-1319.79)	66 (5.8)	0.85 (0.61 ~ 1.19)		1.19 (0.77 ~ 1.72)		1.10 (0.72 ~ 1.67)	
Tertile 3 (1395.77-1809.85)	99 (8.7)	1.29 (0.95 ~ 1.76)	0.078*	1.79 (1.00 ~ 2.97)	0.001*	1.59 (0.95 ~ 2.71)	0.056*
*Per SD increase*							
Cumulative TyG-CVAI	232 (6.8)	1.48 (1.31 ~ 1.68)	< 0.001	1.43 (1.26 ~ 1.62)	< 0.001	1.31 (1.14 ~ 1.52)	< 0.001
Cumulative TyG-BRI	232 (6.8)	1.38 (1.22 ~ 1.55)	< 0.001	1.43 (1.25 ~ 1.62)	< 0.001	1.29 (1.11 ~ 1.49)	< 0.001
Cumulative TyG-CI	232 (6.8)	1.34 (1.18 ~ 1.52)	< 0.001	1.31 (1.15 ~ 1.49)	< 0.001	1.18 (1.02 ~ 1.36)	0.024
Cumulative TyG-WWI	232 (6.8)	1.31 (1.16 ~ 1.49)	< 0.001	1.30 (1.13 ~ 1.50)	< 0.001	1.16 (1.00 ~ 1.35)	0.056
Cumulative TyG-ABSI	232 (6.8)	1.29 (1.14 ~ 1.47)	< 0.001	1.24 (1.08 ~ 1.41)	0.002	1.12 (0.98 ~ 1.29)	0.099
CumulativeTyG-RFM	232 (6.8)	1.16 (1.02 ~ 1.32)	0.025	1.64 (1.26 ~ 2.12)	< 0.001	1.41 (1.08 ~ 1.86)	0.013
*TyG-CVAI longitudinal change pattern*							
Cluster1	48 (3.9)	reference		reference		reference	
Cluster2	95 (6.6)	1.70 (1.18 ~ 2.43)	0.004	1.68 (1.17 ~ 2.42)	0.005	1.54 (1.06 ~ 2.24)	0.022
Cluster3	89 (11.9)	3.08 (2.15 ~ 4.39)	< 0.001	2.83 (1.97 ~ 4.06)	< 0.001	2.28 (1.54 ~ 3.39)	< 0.001
*TyG-BRI longitudinal change pattern*							
Cluster1	58 (4.5)	reference		reference		reference	
Cluster2	95 (6.8)	1.48 (1.07 ~ 2.06)	0.018	1.63 (1.16 ~ 2.28)	0.004	1.44 (1.01 ~ 2.03)	0.041
Cluster3	79 (11.1)	2.36 (1.68 ~ 3.32)	< 0.001	2.61 (1.82 ~ 3.75)	< 0.001	2.01 (1.35 ~ 2.99)	< 0.001
*TyG-CI longitudinal change pattern*							
Cluster1	45 (3.8)	reference		reference		reference	
Cluster2	119 (7.9)	1.55 (1.10 ~ 2.18)	< 0.013	1.55 (1.10 ~ 2.19)	0.014	1.39 (0.97 ~ 1.98)	0.070
Cluster3	68 (9.7)	2.29 (1.61 ~ 3.27)	< 0.001	2.20 (1.53 ~ 3.17)	< 0.001	1.71 (1.16 ~ 2.52)	0.007
*TyG-WWI longitudinal change pattern*							
Cluster1	51 (4.6)	reference		reference		reference	
Cluster2	106 (7.1)	1.48 (1.06 ~ 2.08)	0.021	1.52 (1.07 ~ 2.14)	0.017	1.33 (0.94 ~ 1.88)	0.109
Cluster3	75 (9.5)	2.01 (1.41 ~ 2.86)	< 0.001	1.98 (1.35 ~ 2.89)	< 0.001	1.47 (0.98 ~ 2.21)	0.054
*TyG-ABSI longitudinal change pattern*							
Cluster1	57 (4.9)	reference		reference		reference	
Cluster2	103 (6.9)	1.28 (0.91 ~ 1.80)	0.150	1.24 (0.88 ~ 1.74)	0.220	1.13 (0.80 ~ 1.59)	0.503
Cluster3	72 (9.6)	2.02 (1.43 ~ 2.84)	< 0.001	1.82 (1.27 ~ 2.60)	0.001	1.42 (0.98 ~ 2.07)	0.055
*TyG-RFM longitudinal change pattern*							
Cluster1	74 (6.0)	reference		reference		reference	
Cluster2	63 (5.8)	0.96 (0.68 ~ 1.35)	0.810	1.42 (0.95 ~ 2.11)	0.085	1.20 (0.79 ~ 1.82)	0.388
Cluster3	95 (8.7)	1.25 (0.92 ~ 1.69)	0.158	2.02 (1.10 ~ 3.63)	0.002	1.74 (0.93 ~ 3.24)	0.081

Model I unadjusted. Model II adjusted for age and sex. Model III adjusted for age, sex, smoking, drinking, SBP, DBP, marital status, hukou status, education, HDL-C, LDL-C, uric acid, hemoglobin, eGFR, hypertension, diabetes, CKD, chronic lung disease, antihypertensive agents, antidiabetic agents and antihyperlipidemic agents.*P for trend.

Longitudinal change analysis further highlighted the impact of sustained metabolic status. Stroke incidence increased progressively from the low-level to high-level clusters for six indices (Table S6). Compared with the persistently low-level group (cluster 1), the persistently high-level group (cluster 3) had a significantly elevated risk, with the strongest association for TyG-CVAI (HR 2.28, 95% CI 1.54–3.39), followed by TyG-BRI (2.01, 1.35–2.99) and TyG-CI (1.71, 1.16–2.52). Notably, the moderate-level group (cluster 2) for TyG-CVAI (1.54, 1.06–2.24) and TyG-BRI (1.44, 1.01–2.03) also showed a clear association with increased risk. For TyG-WWI and TyG-ABSI, the persistently high-level group showed borderline associations, whereas no significant risk gradient was observed across change groups for TyG-RFM (Table 3).

Taken together, TyG-CVAI and TyG-BRI demonstrated statistically robust associations across all three analytical dimensions. TyG-CI, TyG-WWI and TyG-ABSI showed relatively weaker and less consistent associations, while TyG-RFM was not statistically significant in most analyses.

### Dose-response relationships between novel TyG-obesity indices and stroke

**Fig. 4:**
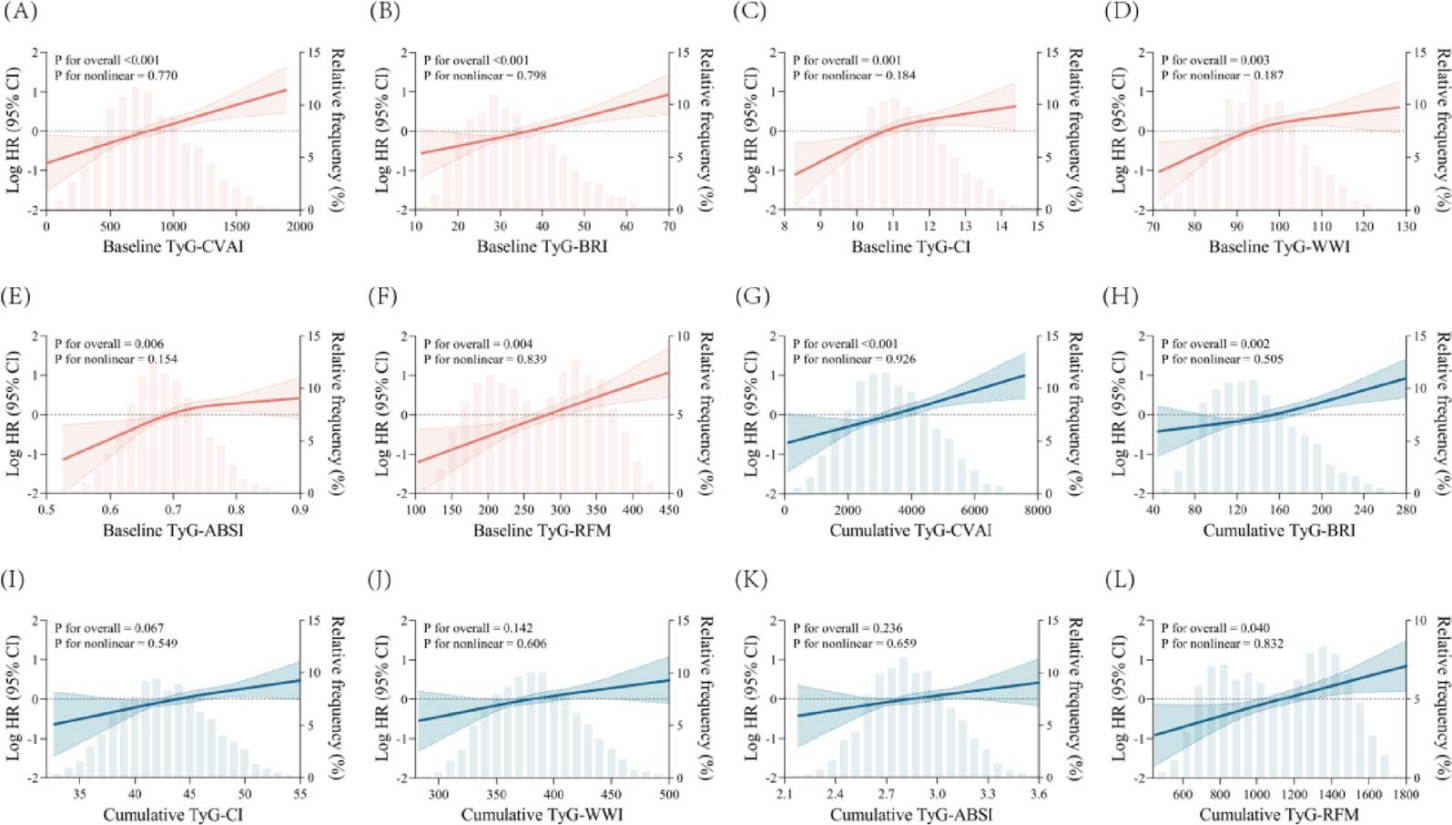
Dose-response associations between baseline/cumulative TyG-obesity indices and stroke in CKM syndrome stages 0–3. **A**–**F** Baseline indices. **G**–**L** Cumulative indices. Models were adjusted for age, sex, smoking, drinking, SBP, DBP, marital status, hukou status, education, HDL-C, LDL-C, uric acid, hemoglobin, eGFR, hypertension, diabetes, CKD, chronic lung disease, antihypertensive, antidiabetic and antihyperlipidemic agents.

RCS analyses generally indicated linear dose-response patterns. Both baseline and cumulative TyG-CVAI and TyG-BRI showed stable, monotonic, and positive risk gradients across observed ranges. Baseline levels of the remaining indices were also positively associated with stroke risk. In cumulative exposure analyses, TyG-RFM showed a statistically significant relationship, TyG-CI exhibited a borderline trend, while the curves for TyG-WWI and TyG-ABSI did not reach statistical significance (Fig. 4).

### Novel TyG-obesity indices for stroke prediction

**Fig. 5:**
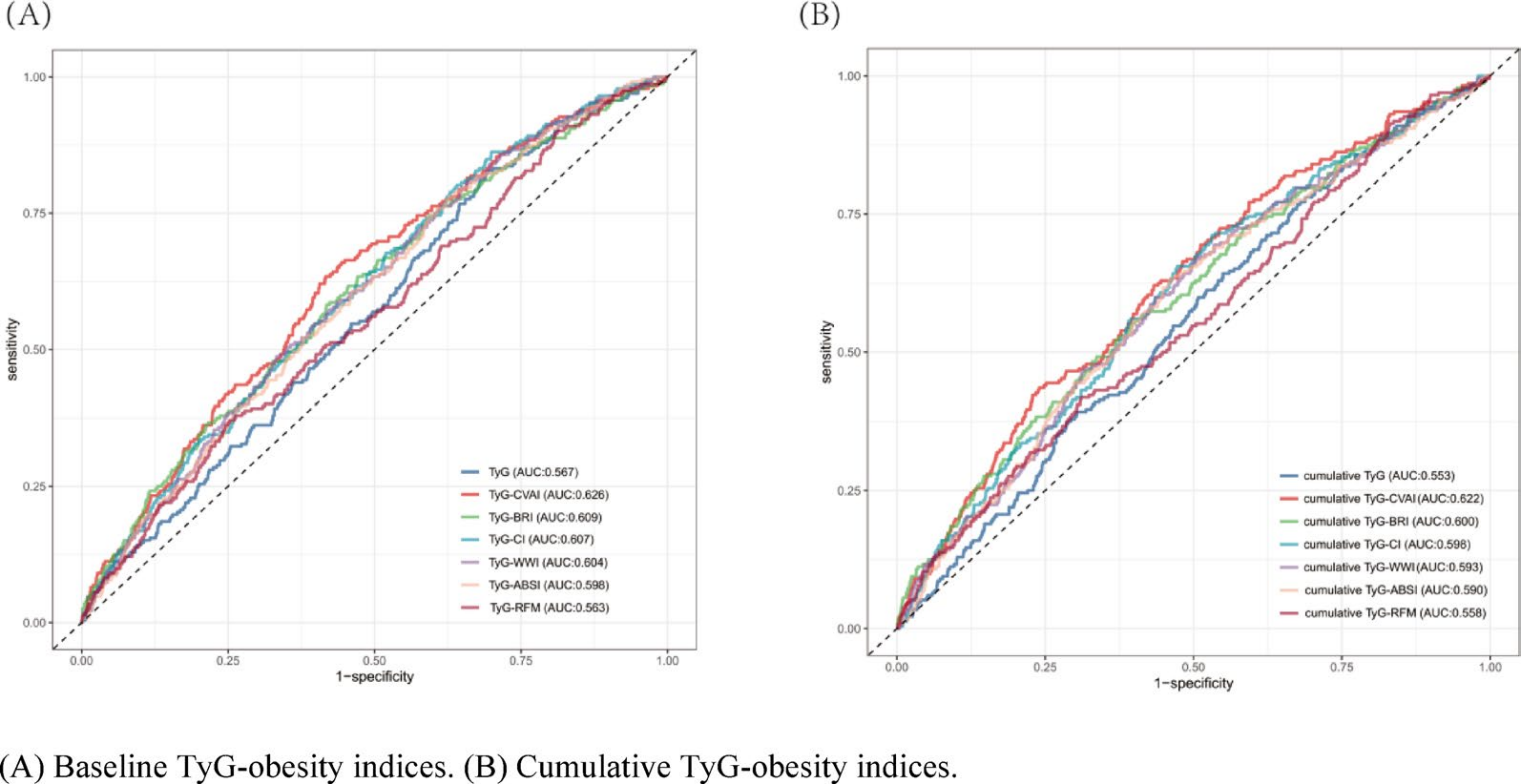
ROC analysis of baseline/cumulative TyG-obesity indices in predicting stroke. **A** Baseline TyG-obesity indices. **B** Cumulative TyG-obesity indices

ROC analysis assessed the predictive performance of the six indices for stroke. TyG-CVAI showed relatively better discrimination, with an AUC of 0.626 (95% CI 0.590–0.663) for baseline levels and 0.622 (95% CI 0.585–0.659) for cumulative exposure. It was followed by TyG-BRI (baseline 0.609, 95% CI 0.573–0.645; cumulative 0.600, 95% CI 0.561–0.639), as well as TyG-CI, TyG-WWI, and TyG-ABSI, each yielding AUCs higher than that of the standalone TyG index. In contrast, TyG-RFM showed the weakest discrimination, with no significant improvement over the TyG index alone (Fig. 5 and Table S7). Based on the basic model comprising conventional risk factors, we evaluated the incremental predictive value of TyG-CVAI and TyG-BRI using the continuous NRI and IDI. Both indices showed statistically significant improvements in risk reclassification (NRI > 0, all *P* < 0.05) and discrimination (IDI > 0, all *P* < 0.05), suggesting that they offer modest incremental information beyond that provided by conventional risk factors (Table S8). DCA indicted that adding TyG-CVAI or TyG-BRI to the basic model yielded a modest net benefit across a range of clinically relevant threshold probabilities, indicating their potential utility for identifying individuals at high risk of stroke (Figs. S3 and S4).

### Associations between TyG-CVAI/TyG-BRI and stroke across CKM stage

**Fig. 6:**
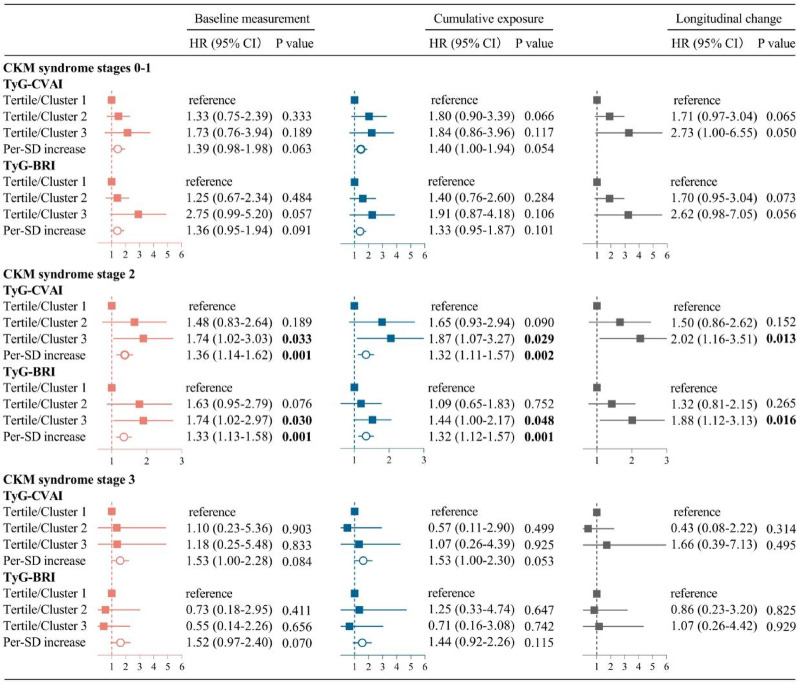
Association between TyG-CVAI/TyG-BRI and stroke across CKM stage. Models were adjusted for age, sex, smoking, drinking, SBP, DBP, marital status, hukou status, education, HDL-C, LDL-C, uric acid, hemoglobin, eGFR, hypertension, diabetes, CKD, chronic lung disease, antihypertensive, antidiabetic and antihyperlipidemic agents

**Fig. 7:**
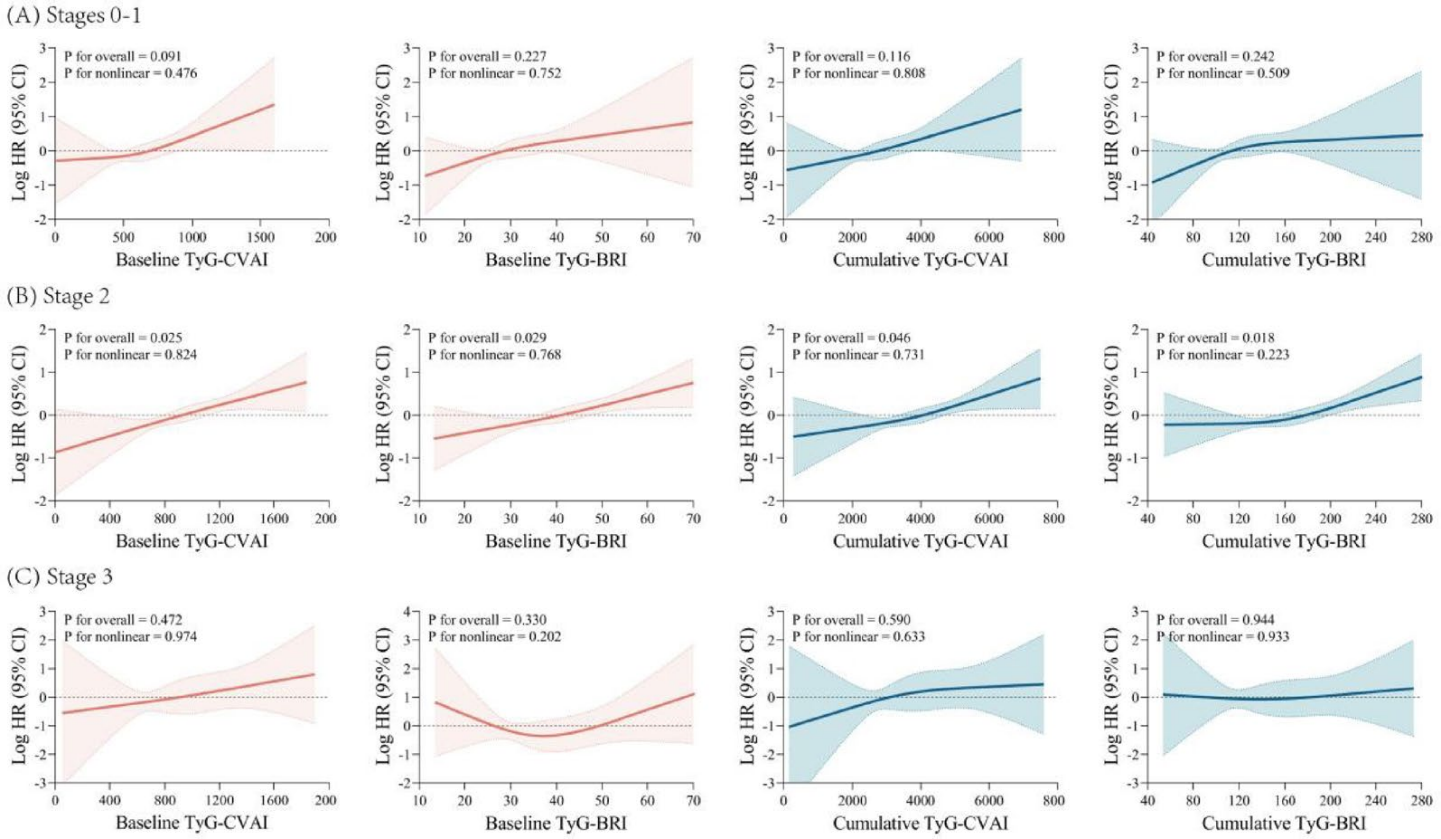
Dose-response relationships between TyG-CVAI/TyG-BRI and stroke across CKM stage. **A** CKM stages 0–1. **B** CKM stage 2. **C** CKM stage 3. Models were adjusted for age, sex, smoking, drinking, SBP, DBP, marital status, hukou status, education, HDL-C, LDL-C, uric acid, hemoglobin, eGFR, hypertension, diabetes, CKD, chronic lung disease, antihypertensive, antidiabetic and antihyperlipidemic agents.

Because TyG-CVAI and TyG-BRI were consistently identified as the stronger predictors in Cox regression, RCS, and ROC analyses, we used these two indices for stage-specific evaluation. Across multidimensional analyses, both indices showed more pronounced associations with stroke in stage 2. For TyG-CVAI, each SD increase in baseline and cumulative exposure was associated with a 36% and 32% higher risk of stroke, and participants in the persistently high-level group had a more than two-fold increased risk (HR 2.02, 95% CI 1.16–3.51). Similar patterns were observed for TyG-BRI, with the persistently high-level group showing an 88% increase in hazard (HR 1.88, 95% CI 1.12–3.13). In contrast, associations in stages 0–1 and stage 3 were non-significant or only marginally significant (Fig. 6). Consistent with these findings, RCS analyses revealed a positive linear association between these two indices and stroke risk in stage 2, whereas no significant trend was observed in other stages (Fig. 7).

### Mediation analyses

Mediation analyses showed that arterial stiffness, as measured by ePWV, partially mediated the relationships between novel TyG-obesity indices and subsequent stroke. The proportion of the total effect mediated by ePWV ranged from 12.8% to 18.0% across different metrics. Specifically, for baseline levels, the mediated proportions were 13.8% for TyG-CVAI, 14.6% for TyG-BRI, 13.5% for TyG-CI, 14.3% for TyG-WWI, 15.7% for TyG-ABSI, and 17.0% for TyG-RFM. When cumulative measurements were analyzed, the mediated proportions were generally similar or slightly higher, including 14.3% for TyG-CVAI, 14.8% for TyG-BRI, 14.4% for TyG-CI, 18.0% for TyG-WWI, 16.3% for TyG-ABSI, and 17.0% for TyG-RFM (Fig. S5).

### Sensitivity analyses

Sensitivity analyses supported the robustness of the primary findings. First, excluding participants who experienced stroke during wave 4 did not materially change the results, with TyG-CVAI and TyG-BRI remaining the stronger predictors (Tables S9 and S10, Fig. S6). Second, stratified analyses of these two indices revealed consistent associations across all subgroups, with no significant effect modification observed (all interaction *P* > 0.05). After controlling for the false discovery rate, the absence of interactions remained consistent (all q-values > 0.05) (Figures S7 and S8, Table S11). Third, the Fine-Gray subdistribution hazard model, accounting for all-cause mortality as a competing event, did not materially alter the associations of TyG-CVAI and TyG-BRI with incident stroke (Tables S12 and S13).

## Discussion

In this nationwide prospective cohort study, six novel TyG-obesity indices were differentially associated with incident stroke in individuals with early-stage CKM syndrome. Among these, TyG-CVAI and TyG-BRI consistently showed stronger and more robust associations across baseline, cumulative, and longitudinal change-based analyses. Their predictive performance compared favorably with that of the standalone TyG index and the other composite measures. Notably, the associations of these two indices with stroke were more pronounced in CKM stage 2, a metabolically active phase representing the largest subgroup. These findings suggest that integrating IR with measures of pathogenic fat distribution, as captured by TyG-CVAI and TyG-BRI, may offer a promising approach to stroke risk stratification in early-stage CKM syndrome, particularly in stage 2, where they could facilitate more refined, stage-matched monitoring.

Accumulating evidence supports the utility of IR surrogates combined with anthropometric metrics for assessing adverse cardio-cerebrovascular outcomes [8, 27, 28]. Integrating the TyG index with traditional obesity indices (e.g., TyG-BMI, TyG-WC, TyG-WHtR) typically yields superior predictive performance compared to the original index, underscoring the rationale for jointly evaluating IR and adiposity [23, 29]. However, traditional anthropometric measures possess inherent limitations. BMI does not distinguish lean mass from adipose tissue, WC is not normalized for height, and WHtR, while an improvement, remains a simplified geometric proxy. Advances in obesity phenotyping have introduced novel anthropometric indices that employ complex mathematical algorithms to estimate body shape and volume, thereby providing a more accurate reflection of visceral fat accumulation and associated metabolic risks [30–33]. Visceral obesity is significantly associated with the development of IR, systemic inflammation, and oxidative stress, all of which contribute to the progression of the CKM continuum [34]. Recent studies based on cross-sectional or baseline data have reported that novel anthropometry-based TyG extensions show promise for stroke prediction [35–37]. For instance, TyG-ABSI demonstrated a positive and non-linear association with stroke in middle-aged and older adults [38], and TyG-BRI effectively predicted ischemic stroke in susceptible populations [39]. A comparative analysis of four indices (TyG-BRI, TyG-ABSI, TyG-WWI, and TyG-CVAI) indicated that TyG-CVAI exhibited incremental predictive value for stroke in individuals with CKM stages 0–3 [15]. Longitudinal studies further revealed that cumulative TyG-CVAI exposure was independently associated with early stroke risk in the general population [37], and that TyG-CVAI ranked among the top predictors for cardiovascular disease in a trajectory analysis encompassing eight TyG-derived indicators [23].

Despite these advances, important evidence gaps remain. Most existing studies have relied on baseline measurements alone, failing to capture the cumulative burden and dynamic evolution of metabolic risk over time. Available longitudinal analyses have been confined to single or limited indicators or conducted across heterogeneous populations and outcomes. Consequently, systematic comparisons of novel TyG-obesity indices in the metabolically vulnerable CKM population are lacking, hindering the identification of more informative indicators for long-term monitoring. The present investigation addresses these gaps through multidimensional analyses that integrate baseline and longitudinal assessments to capture both cross-sectional and temporal dimensions of exposure, incorporate a broader panel of indices to enable comprehensive comparisons, furthermore, delineate the specific stage-specific window in which these indices may exert better predictive utility. These observations extend previous work and offer new insights into the role of metabolic obesity phenotypes in stroke-prone populations.

Through baseline analysis, we reaffirmed the positive associations between novel TyG-obesity indices and incident stroke. Standardized comparisons revealed heterogeneity in the strength of these associations. TyG-CVAI and TyG-BRI exhibited more robust associations, consistent with findings from a recent four-index study [15]. Notably, TyG-CI and TyG-RFM have both been previously reported as effective predictors of cardiovascular risk in general populations [40, 41], yet they exhibited distinct patterns in the current study. TyG-CI showed a significant but weaker association, whereas TyG-RFM did not demonstrate a stable relationship with stroke. These differences suggest that the predictive utility of specific indices may depend on the underlying pathophysiological context, particularly pre-existing metabolic dysregulation and cardiorenal comorbidities. Therefore, clinical risk assessment may benefit from using indicators tailored to distinct population characteristics.

Cumulative exposure analyses further confirmed the robustness of TyG-CVAI and TyG-BRI as stronger indicators, whereas other composites showed inconsistent or marginal associations. Paralleling these findings, longitudinal change pattern analyses revealed a clear and graded pattern of stroke risk for TyG-CVAI and TyG-BRI. Compared with the stable low-level group, the sustained high-level group was associated with a 2.3-fold and 2.0-fold increase in hazard, respectively. Even the moderate-level group conferred significantly elevated risks of 54% and 44%. This stepwise risk pattern suggests that these two indices may capture subtle variations in risk, moving beyond a simple high-versus-low dichotomy and enabling more refined stratification. In contrast, TyG-CI showed elevated risk only in the persistent high-level group, indicating limited discriminatory ability. A previous study in early-stage CKM syndrome reported that persistently high TyG levels were not significantly associated with stroke risk, a finding potentially attributable to limited statistical power, adaptive physiological responses to metabolic stress, or intensive medical management [42]. In contrast, our findings demonstrate that sustained exposure to an unfavorable metabolic profile, quantified by integrated measures of IR and visceral adiposity, is associated with a stepwise increase in stroke risk. This discrepancy highlights the added value of jointly assessing metabolic and obesity-related pathways beyond evaluating IR in isolation, and aligns with the broader literature emphasizing the importance of dynamic monitoring for early identification of high-risk individuals [43–46]. These observations suggest that both the magnitude and persistence of elevated TyG-CVAI and TyG-BRI contribute to stroke incidence, reinforcing the potential of interventions aimed at reducing or stabilizing these indices in primary prevention.

Previous stage-specific studies have yielded inconsistent findings. For instance, a NHANES-based analysis reported significant associations of TyG-related indices with all-cause and cardiovascular mortality in CKM stages 1 and 3, but not in stages 0 or 2 [17]. A CHARLS study found that TyG-ABSI had greater predictive value for mortality in advanced CKM syndrome (stages 3–4) [47]. These discrepancies likely reflect heterogeneity in CKM staging criteria, exposure definitions, outcome measures, and population characteristics across studies. In our study, the associations between TyG-CVAI/BRI and stroke were more pronounced in CKM stage 2, a phase where metabolic dysregulation may act as a primary driver of vascular injury, preceding the development of subclinical (stage 3) or overt CVD(stage 4). Indices that integrate metabolic and anthropometric profiles may be more informative at this stage because they directly reflect active metabolic disturbances. This pattern differs from a prior study that reported a significant association between cumulative TyG level and stroke risk only in stage 3 [42]. Notably, that study relied on traditional risk assessment tools to define subclinical CVD, resulting in a disproportionately high proportion of stage 3 participants (53.9%) compared with stage 2 (29.6%). In contrast, we used the AHA-recommended PREVENT model [48], which yielded a more pragmatic stage distribution (51.3% for stage 2, and 9.6% for stage 3), aligning with recent nationwide epidemiological data from South Korea (43.4% for stage 2, and 7.3% for stage 3) [49]. Our stage-specific analysis incorporated dynamic assessments, overcoming the limitations of single-timepoint measurements and enabling monitoring of TyG-CVAI/BRI changes that may better reflect the impact of metabolic control status on progression along the CKM continuum. These findings highlight the potential public health relevance of applying these risk markers for early detection and intervention in the large, asymptomatic subgroup of stage 2. The attenuated associations in stages 0–1 likely reflect lower baseline risk, whereas in stage 3 the predictive value may be diluted by established subclinical disease, competing clinical events, or intensive pharmacological interventions. In addition, the wide confidence intervals in stage 3 due to limited sample size warrant cautious interpretation and validation in larger, well-powered cohorts.

CKM syndrome is a prevalent multisystem disorder characterized by the convergence of multiple pathophysiological pathways, including IR, lipotoxicity, oxidative stress, and systemic inflammation. The observed associations may reflect the synergistic effects of IR and visceral adiposity, which are considered mechanistic cornerstone of the CKM continuum [5]. IR promotes the formation of advanced glycation end products (AGEs), which can activate NF-κB and NLRP3 inflammasome pathways, leading to the transcription of pro-inflammatory cytokines such as TNF-α, IL-6, and IL-1β. AGEs also stimulate NADPH oxidase, contributing to reactive oxygen species generation and persistent oxidative stress that may impair mitochondrial function [50, 51]. These processes have been linked to vascular senescence, endothelial disruption, and impaired vascular homeostasis. Endothelial dysfunction, in turn, facilitates monocyte recruitment, lipid retention, and foam cell formation, potentially accelerating atherogenesis [52]. Concurrently, dysfunctional visceral adipose tissue releases free fatty acids, pro-inflammatory factors, and oxidative stress mediators, while reducing adiponectin expression. This can lead to lipotoxicity, chronic inflammation, and impaired insulin signaling, exacerbating IR and establishing a self-perpetuating cycle [53]. This cycle may contribute to hypertension, pro-thrombotic state, progressive endothelial dysfunction, and increased atherosclerotic burden, ultimately elevating stroke risk. Mediation analysis suggested that arterial stiffness, as estimated by ePWV, partially mediated the association between the adverse metabolic obesity phenotype and elevated stroke risk. This finding is biologically plausible, as vascular stiffening and cerebral atherosclerosis share common pathophysiological substrates, including IR, chronic low-grade inflammation, oxidative stress, and endothelial dysfunction. Given that ePWV is a mathematical surrogate derived from age and blood pressure, future studies incorporating direct measures of arterial stiffness (e.g., carotid-femoral pulse wave velocity) are needed to further evaluate this mediation.

The AHA designates individuals with CKM stages 0–3 as a priority group for early risk stratification, highlighting the need for reliable indicators to detect latent metabolic dysregulation before overt organ damage occurs. Integrating TyG with CVAI and BRI may facilitate stroke risk assessment, whether measured at a single time point or tracked longitudinally. This approach appears particularly relevant for CKM stage 2, the largest subgroup of asymptomatic individuals. Moreover, the required parameters are routinely available even in resource-limited settings, supporting their feasibility as screening tools. It should be noted, however, that as with all metabolic biomarkers, the components of these indices overlap with CKM staging criteria, potentially introducing tautology or mathematical coupling. Therefore, future validation in cohorts using non-overlapping staging criteria (e.g., imaging-defined subclinical CVD) or in populations with different anthropometric profiles is needed to determine whether they offer additional pathophysiological insights beyond this inherent coupling.

### Strengths and limitations

The strengths of this study include a head-to-head comparison of six novel indices, integration of static and dynamic assessments, use of the Boruta algorithm to objectively rank the importance of features, and identification of stage-specific heterogeneity. Several limitations should be acknowledged. First, the observational design precludes causal inferences. Second, despite adjustment for a wide range of confounders, residual confounding cannot be entirely ruled out. Third, stroke events were identified based on self-reported physician diagnoses rather than imaging (CT/MRI), which may introduce recall bias or misclassification [54]. However, recent validation studies suggest limited impact of such biases [55]. Fourth, because blood samples were collected only at waves 1 and 3, more sophisticated trajectory methods (e.g., latent class growth analysis) could not be applied. Future studies with greater longitudinal depth (e.g., multiple blood collections) should employ such growth‑modeling approaches. Fifth, in stage-specific analyses, the limited sample size and absence of direct imaging or biomarker evidence for subclinical CVD may have led to some misclassification between stages 2 and 3. Therefore, the findings require validation in larger cohorts with comprehensive subclinical disease assessment. Sixth, baseline differences between included and excluded participants (standardized differences > 0.1 for some variables) may introduce selection bias (Table S14), potentially limiting generalizability to broader CKM populations. However, exclusion was strictly based on data availability rather than outcome status, which substantially reduces this risk. Finally, the study included only middle-aged and older Chinese adults, limiting generalizability to other populations. All findings should be further validated in independent, diverse cohorts before clinical application.

## Conclusions

Among the six novel TyG-obesity indices evaluated, differential associations with incident stroke were observed in individuals with early-stage CKM syndrome, with TyG-CVAI and TyG-BRI showing stronger and more robust associations, particularly in CKM stage 2. These findings suggest that these two indices may serve as promising tools for refined risk stratification and early prevention in this growing at-risk population.

## Supplementary Information

Below is the link to the electronic supplementary material.

Supplementary Material 1.

Supplementary Material 2.

## Data Availability

CHARLS datasets are available for download at the CHARLS home website (https://charls.pku.edu.cn/en/).

## Abbreviations

CKM syndrome: Cardiovascular–kidney–metabolic syndrome
CHARLS: China Health and Retirement Longitudinal Study
IR: Insulin resistance
CVD: Cardiovascular disease
TyG-CVAI: Triglyceride-glucose Chinese visceral adiposity index
TyG-BRI: Triglyceride-glucose body roundness index
TyG-CI: Triglyceride-glucose conicity index
TyG-WWI: Triglyceride-glucose weight-adjusted waist index
TyG-ABSI: Triglyceride-glucose a body shape index
TyG-RFM: Triglyceride-glucose relative fat mass
TyG-BMI: Triglyceride-glucose body mass index
TyG-WC: Triglyceride-glucose waist circumference
TyG-WHtR: Triglyceride-glucose waist-to-height ratio
eGFR: Estimated glomerular filtration ratio
LDL-C: Low density lipoprotein cholesterol
HDL-C: High density lipoprotein cholesterol
CKD: Chronic kidney disease
