# Supplementary material for: Differential associations between novel triglyceride-glucose-obesity indices and incident stroke in individuals with early-stage cardiovascular-kidney-metabolic syndrome: a longitudinal cohort study
Source: Cardiovasc Diabetol. 2026 Jun 8;25:216. doi: 10.1186/s12933-026-03240-x (PMC13428451; doi:10.1186/s12933-026-03240-x)
Supplement: Supplementary file 1 — Supplementary Material 1. [file 12933_2026_3240_MOESM1_ESM.docx]

**Supplemental Material 1**

**Differential associations between novel triglyceride-glucose-obesity indices and incident stroke in individuals with early-stage cardiovascular-kidney-metabolic syndrome: a longitudinal cohort study**

Zhi-Qing Fu, Li An, Wei Zhang, Yue Lyu, Shan Li*

**Table S1.** The definitions of various diseases

**Table S2.** Definition of CKM syndrome staging adjusted for data in CHARLS

**Table S3.** The basic PREVENT 10-year risk estimation model equations

**Table S4.** Calculation formula

**Table S5.** The levels of the TyG index and obesity indices at waves 1 and 3

**Figure S1.** The elbow plot for K-means algorithm

**Figure S2.** Evolution of the number of iterations in the Boruta algorithm

**Table S6.** The incidence of stroke among different TyG-obesity trajectory groups

**Table S7.** Predictive performance of TyG-obesity indices for stroke by ROC analysis

**Table S8.** Incremental predictive value of baseline, cumulative, and longitudinal change in TyG-CVAI and TyG-BRI for stroke in CKM syndrome stage 0-3

**Figure S3.** Decision curve analysis for comparing TyG‑CVAI and basic model in stroke prediction in CKM syndrome stages 0-3

**Figure S4.** Decision curve analysis for comparing TyG‑BRI and basic model in stroke prediction in CKM syndrome stages 0-3

**Figure S5.** Path diagram of the mediation analysis of ePWV

**Table S9.** Association between baseline TyG-obesity indices and stroke in CKM syndrome stages 0-3: Excluding participants with stroke at wave 4 (n=3,278)

**Table S10.** Association between cumulative exposure and longitudinal trajectories in TyG-obesity indices and stroke in CKM syndrome stages 0-3: Excluding participants with stroke at wave 4

**Figure S6.** Restricted cubic spline curve for the association between TyG-obesity indices and new-onset stroke : Excluding participants with stroke at wave 4 (n=3,278)

**Figure S7**. Subgroup analyses of associations between baseline TyG-CVAI/BRI and stroke

**Figure S8**. Subgroup analyses of associations between cumulative TyG-CVAI/BRI and stroke

**Table S11.** Subgroup analyses of associations between longitudinal change of TyG-CVAI/BRI and incident stroke

**Table S12.** Associations of baseline, cumulative, and longitudinal change in TyG-CVAI with stroke in CKM syndrome stages 0-3: A Fine‑Gray competing risks analysis

**Table S13.** Associations of baseline, cumulative, and longitudinal change in TyG-BRI with stroke in CKM syndrome stages 0-3: A Fine‑Gray competing risks analysis

**Table S14.** Comparison of baseline characteristics of included participants and those excluded due to missing TyG-related indices and CKM staging

**Table S1. The definitions of various diseases**

| Prediabetes | Fasting blood glucose ≥ 100-125 mg/dL or HbA1c ≥ 5.7%-6.4% and without self-reported diagnosis of diabetes, use of insulin, or oral hypoglycemic agents |
| --- | --- |
| Diabetes | Fasting blood glucose ≥ 126 mg/dL or HbA1c ≥ 6.5% or self-reported diagnosis of diabetes, use of insulin, or oral hypoglycemic agents |
| Hypertension | SBP ≥140 mm Hg or DBP ≥90 mm Hg or self-reported diagnosis of hypertension or use of antihypertensive medications |
| Chronic kidney disease | Any of the two criteria is met：  ① Self-reported history of chronic kidney disease  ② eGFR < 60 ml/min/1.73m^2^ |
| Hypertriglyceridemia | Triglycerides ≥ 135 mg/dL |
| Metabolic syndrome | Meet any three or more of the five  ① Waist circumference ≥ 80/90 cm in female/male  ② HDL cholesterol <50/40 mg/dL in female/male  ③ Triglycerides ≥150 mg/dL  ④ Elevated blood pressure (SBP ≥130 mm Hg or DBP ≥80 mm Hg and/or use of antihypertensive medications)  ⑤ Fasting blood glucose ≥100 mg/dL |
| Cardiovascular disease | ① self-reported diagnosis of heart disease  ② or self-reported diagnosis of stroke |

**Table S2. Definition of CKM syndrome staging adjusted for data in CHARLS**

| **Stages** | **Definition** |
| --- | --- |
| Stage 0: No CKM risk factors | Individuals who meet all of the following criteria: 1. Not overweight or obese (BMI < 23 kg/m^2^; waist circumference < 80/90 cm for women/men). 2. Absence of metabolic risk factors such as hypertension, hypertriglyceridemia (<135 mg/dL), metabolic syndrome, prediabetes, diabetes, or chronic kidney disease (CKD). |
| Stage 1: Excess adiposity or dysfunctional metabolism | Individuals with a BMI ≥ 23 kg/m^2^, or a waist circumference ≥ 80/90 cm for women/men, and/or prediabetes, but without additional metabolic risk factors or CKD. |
| Stage 2: Presence of metabolic risk factors and CKD | Individuals who exhibit at least one metabolic risk factor such as hypertriglyceridemia (≥135 mg/dL), hypertension, metabolic syndrome, diabetes, or have been diagnosed with CKD. |
| Stage 3: Subclinical Cardiovascular Disease (CVD) in CKM | 1. Subclinical ASCVD or subclinical HF among individuals with excess/dysfunctional adiposity, other   metabolic risk factors, or CKD   1. Risk equivalents of subclinical CVD*   High predicted 10-y CVD risk (20% or higher, based on the PREVENT model)  Very high-risk CKD (G4 or G5 CKD or very high risk per KDIGO classification) |
| Stage 4: Clinical CVD in CKM | Individuals have been diagnosed with clinical CVD (heart disease or stroke) |

CKM syndrome, Cardiovascular-Kidney-Metabolic syndrome. CHARLS, China Health and Retirement Longitudinal Study. CKD, chronic kidney disease. CVD, cardiovascular disease. ASCVD，atherosclerotic cardiovascular disease.

*Due to the lack of relevant diagnostic data on subclinical CVD, such as coronary catheterisation, computed tomography angiography, echocardiography or cardiac biomarkers, we defined subclinical CVD using risk equivalents. This included individuals with a predicted 10-year CVD risk of 20% or higher, based on the PREVENT (Predict Risk of Cardiovascular Disease EVENTs) model, or those with very high-risk CKD at Stages G4 or G5.

Reference

1. KDIGO 2024 Clinical Practice Guideline for the Evaluation and Management of Chronic Kidney Disease. Kidney international. 2024;105(4s):S117-s314.

2. Inker LA, Eneanya ND, Coresh J, Tighiouart H, Wang D, Sang Y, et al. New Creatinine- and Cystatin C-Based Equations to Estimate GFR without Race. The New England journal of medicine. 2021;385(19):1737-49.

3. Whelton PK, Carey RM. The 2017 American College of Cardiology/American Heart Association Clinical Practice Guideline for High Blood Pressure in Adults. JAMA cardiology. 2018;3(4):352-3.

4. Classification and Diagnosis of Diabetes: Standards of Medical Care in Diabetes-2022. Diabetes care. 2022;45(Suppl 1):S17-s38.

5. Khan SS, Matsushita K, Sang Y, Ballew SH, Grams ME, Surapaneni A, et al. Development and Validation of the American Heart Association's PREVENT Equations. Circulation. 2024;149(6):430-49.

**Table S3. The basic PREVENT 10-year risk estimation model equations**

| **10-year CVD risk assessment equation** | |
| --- | --- |
| **Men** | **log-Odds** = -3.031168 + 0.7688528 × (age – 55) /10 + 0.0736174 × ((TC – HDL-C) × 0.02586 – 3.5) – 0.0954431 × (HDL-C × 0.02586 – 1.3) /0.3 – 0.4347345 × (min(SBP, 110) – 110) /20 + 0.3362658 × (max(SBP, 110) – 130) /20 + 0.7692857 × (if diabetes) + 0.4386871 × (if current smoker) + 0.5378979 × (min(eGFR, 60) – 60) / -15 + 0.0164827 × (max(eGFR, 60) – 90) / -15 + 0.288879 × (if using anti hypertensive medication) – 0.1337349 × (if using statin) – 0.0475924 × (if using anti-hypertensive medication) × (max(SBP, 110) – 130) /20 + 0.150273 × (if using statin) × ((TC – HDL-C) × 0.02586 – 3.5) – 0.0517874 × (age – 55) /10 × ((TC – HDL-C) × 0.02586 – 3.5) + 0.0191169 × (age – 55) /10 × (HDL-C × 0.02586 – 1.3) /0.3 – 0.1049477 × (age – 55) /10 × (max(SBP, 110) – 130) /20 – 0.2251948 × (age – 55) /10 × (if diabetes) – 0.0895067 × (age – 55) /10 × (if current smoker) – 0.1543702 × (age – 55) /10 × (min(eGFR, 60) – 60) / -15 |
|  | **Risk**= 1 / (1 + exp(-log-Odds)) |
| **Women** | log-Odds = -3.307728 + 0.7939329 × (age – 55) /10 + 0.0305239 × ((TC – HDL-C) × 0.02586 – 3.5) – 0.1606857 × (HDL-C × 0.02586 – 1.3) /0.3 – 0.2394003 × (min(SBP, 110) – 110) /20 + 0.360078 × (max(SBP, 110) – 130) /20 + 0.8667604 × (if diabetes) + 0.5360739 × (if current smoker) + 0.6045917 × (min(eGFR, 60) – 60) / -15 + 0.0433769 × (max(eGFR, 60) – 90) / -15 + 0.3151672 × (if using anti hypertensive medication) – 0.1477655 × (if using statin) – 0.0663612 × (if using anti-hypertensive medication) × (max(SBP, 110) – 130) /20 + 0.1197879 × (if using statin) × ((TC – HDL-C) × 0.02586 – 3.5) – 0.0819715 × (age – 55) /10 × ((TC – HDL-C) × 0.02586 – 3.5) + 0.0306769 × (age – 55) /10 × (HDL-C × 0.02586 × 0.02586 – 1.3) /0.3 – 0.0946348 × (age – 55) /10 × (max(SBP, 110) – 130) /20 – 0.27057 × (age – 55) /10 × (if diabetes) – 0.078715 × (age – 55) /10 × (if current smoker) – 0.1637806 × (age – 55) /10 × (min(eGFR, 60) – 60) / -15 |
|  | **Risk**= 1 / (1 + exp(-log-Odds)) |

TC, total cholesterol; HDL-C, high-density lipoprotein cholesterol; SBP, systolic blood pressure; eGFR, estimated glomerular filtration rate.

**Table S4. Calculation formula**

| **Indicator** | | **Calculation Formula** | |
| --- | --- | --- | --- |
| TyG Index | | TyG Index = ln[TG (mg/dL) × FBG (mg/dL) / 2] | |
| CVAI | Male | CVAI = -67.93 + 0.68×Age (years) + 0.03×BMI (kg/m²) + 4.00×WC (cm) + 22.00×log_10_[TG (mmol/L] - 16.32×HDL-C (mmol/L) | |
|  | Female | CVAI = -187.32 + 1.71×Age (years) + 4.23×BMI (kg/m²) + 1.12×WC (cm) + 39.76×log_10_[TG (mmol/L] - 11.66×HDL-C (mmol/L) | |
| BRI | | BRI= 364.2-365.5×{1-[WC(m)/2π]^2^} / [0.5×height(m)^2^]^1/2^ | |
| CI | | CI = WC (m) / 0.109 × (Weight (kg) / Height (m)^1/2^ | |
| WWI | | WWI= WC(cm) /weight(kg)^1/2^ | |
| ABSI | | ABSI=WC(m)/(BMI^2/3^ $\times$height(m)^1/2^) | |
| RFM | | RFM = 64 - [20 × Height (m) / Waist Circumference (m)], sex= 0 for males and 1 for females | |
| Baseline TyG-CVAI | | TyG-CVAI_2012_ = TyG_2012_×CVAI_2012_ | TyG-CVAI_2015_ = TyG_2015_×CVAI_2015_ |
| Baseline TyG-BRI | | TyG-BRI_2012_ = TyG_2012_×BRI_2012_ | TyG-BRI_2015_ = TyG_2015_×BRI_2015_ |
| Baseline TyG-CI | | TyG-CI_2012_ = TyG_2012_×CI_2012_ | TyG-CI_2015_ = TyG_2015_×CI_2015_ |
| Baseline TyG-WWI | | TyG-WWI_2012_ = TyG_2012_×WWI_2012_ | TyG-WWI_2015_ = TyG_2015_×WWI_2015_ |
| Baseline TyG-ABSI | | TyG-ABSI_2012_ = TyG_2012_×ABSI_2012_ | TyG-ABSI_2015_ = TyG_2015_×ABSI_2015_ |
| Baseline TyG-RFM | | TyG-RFM_2012_ = TyG_2012_×RFM_2012_ | TyG-RFM_2015_ = TyG_2015_×RFM_2015_ |
| Cumulative TyG-CVAI | | Cumulative TyG-CVAI = (TyG-CVAI_2012_ + TyG-CVAI_2015_)/2 ×ΔTime, ΔTime is the time interval between 2012 and 2015 | |
| Cumulative TyG-BRI | | Cumulative TyG-BRI = (TyG-BRI_2012_ + TyG-BRI_2015_)/2 ×ΔTime | |
| Cumulative TyG-CI | | Cumulative TyG-CI = (TyG-CI_2012_ + TyG-CI_2015_)/2 ×ΔTime | |
| Cumulative TyG-WWI | | Cumulative TyG-WWI = (TyG-WWI_2012_ + TyG-WWI_2015_)/2 ×ΔTime | |
| Cumulative TyG-ABSI | | Cumulative TyG-ABSI = (TyG-ABSI_2012_ + TyG-ABSI_2015_)/2 ×ΔTime | |
| Cumulative TyG-RFM | | Cumulative TyG-RFM = (TyG-RFM_2012_ + TyG-RFM_2015_)/2 ×ΔTime | |
| ePWV | | ePWV = 9.587 - 0.402×Age (years) + 4.560×10^-3^×Age (years)^2^ - 2.621×10^-5^×Age (years)^2^×MBP + 3.176×10^-3^×Age (years)× MBP - 1.832×10^-2^×MBP^2^, MBP = DBP + 0.4×(SBP - DBP) | |

TyG, triglyceride-glucose index. WC, waist circumference. CVAI, Chinese visceral adiposity index. BRI, body roundness index. CI, conicity index. ABSI, a body shape index. WWI, weight-adjusted waist index. RFM, relative fat mass. ePWV, estimated pulse wave velocity.

**Table S5. The levels of the TyG index and novel obesity indices at waves 1 and 3**

| **Variables** | **2012** | **2015** |
| --- | --- | --- |
| TyG | 8.59 ± 0.56 | 8.61 ± 0.55 |
| WC, cm | 84.79 ± 9.27 | 85.70 ± 9.74 |
| Height, m | 1.58 ± 0.08 | 1.58 ± 0.08 |
| Weight | 58.56 ± 10.21 | 58.84 ± 10.55 |
| CVAI | 92.53 ± 36.26 | 101.27 ± 37.27 |
| BRI | 4.15 ± 1.25 | 4.31 ± 1.33 |
| CI | 1.28 ± 0.08 | 1.29 ± 0.08 |
| WWI | 11.13 ± 0.79 | 11.22 ± 0.81 |
| ABSI | 0.083 ± 0.005 | 0.083 ± 0.005 |
| RFM | 32.75 ± 8.45 | 33.21 ± 8.61 |

**Figure S1. The elbow plot for K-means algorithm**

**
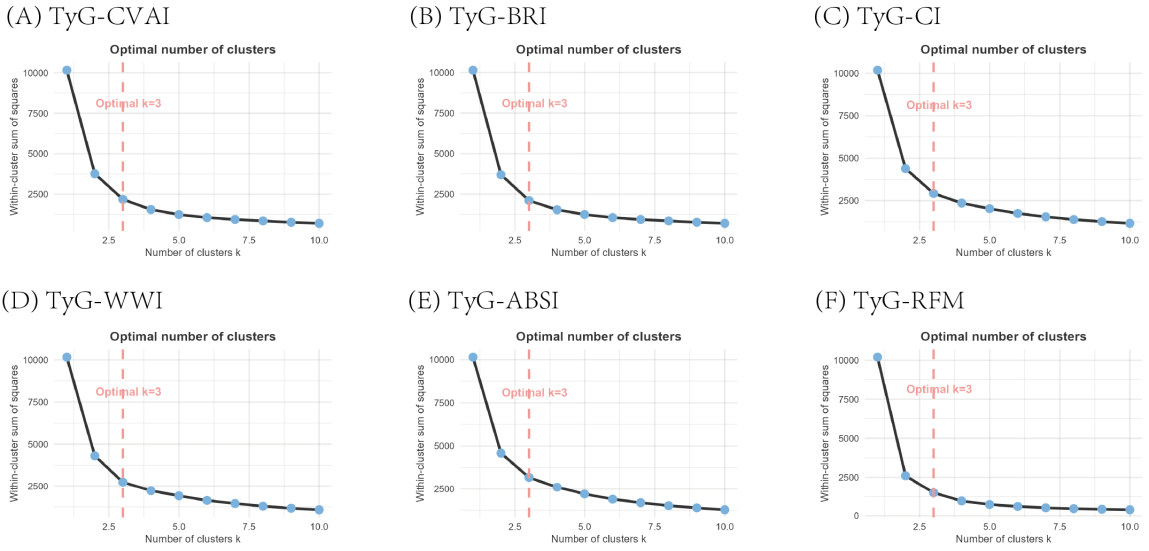
**

**Figure S2. Evolution of the number of iterations in the Boruta algorithm**

1. (B)


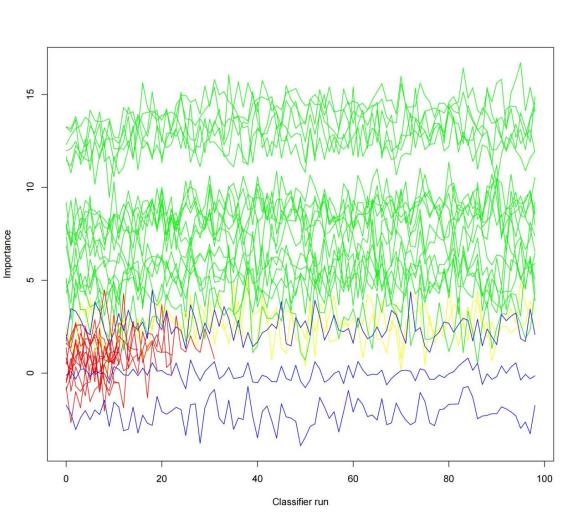

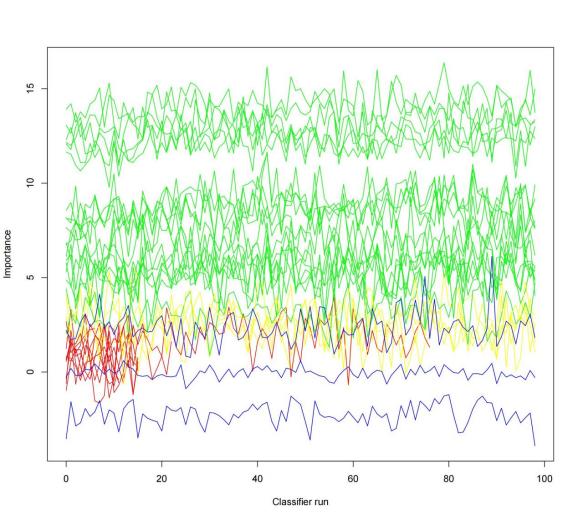


(C)


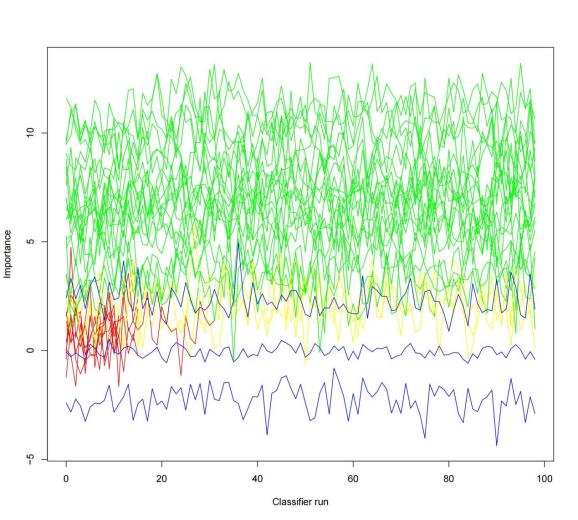


1. Baseline TyG-obesity indices. (B) Cumulative TyG-obesity indices. (C) Longitudinal change patterns of TyG-obesity indices.

The plot shows the importance trajectories of each feature over 100 random forest runs. Green lines indicate confirmed features; yellow lines, tentative features; red lines, rejected features; blue lines represent shadow features.

**Table S6. The incidence of stroke among different TyG-obesity longitudinal change groups**

| Variable | Mean ± SD | | Number  (n, %) | Events  (n, %) | P value |
| --- | --- | --- | --- | --- | --- |
|  | 2012 | 2015 |  |  |  |
| **TyG-CVAI longitudinal change pattern** | | | | | <0.001 |
| Cluster 1 | 461.33 ± 161.99 | 520.28 ± 176.67 | 1160 (34.1) | 44 (3.8) |  |
| Cluster 2 | 824.23 ± 150.07 | 915.82 ± 155.42 | 1411 (41.5) | 92 (6.5) |  |
| Cluster 3 | 1254.57 ± 197.54 | 1335.20 ± 203.04 | 829 (24.4) | 96 (11.6) |  |
| **TyG-BRI longitudinal change pattern** | | | | | <0.001 |
| Cluster 1 | 24.95 ± 4.85 | 25.44 ± 5.36 | 1275 (37.5) | 57 (4.5) |  |
| Cluster 2 | 37.06 ± 5.48 | 39.00 ± 5.62 | 1381 (40.6) | 94 (6.8) |  |
| Cluster 3 | 52.26 ± 7.17 | 54.66 ± 7.67 | 744 (21.9) | 81 (10.9) |  |
| **TyG-CI longitudinal change pattern** | | | | | <0.001 |
| Cluster 1 | 9.96 ± 0.62 | 10.01 ± 0.64 | 1101 (32.4) | 48 (4.4) |  |
| Cluster 2 | 11.07 ± 0.59 | 11.22 ± 0.61 | 1449 (42.6) | 99 (6.8) |  |
| Cluster 3 | 12.33 ± 0.70 | 12.43 ± 0.75 | 850 (25.0) | 85 (10.0) |  |
| **TyG-WWIlongitudinal change pattern** | | | | | <0.001 |
| Cluster 1 | 85.81 ± 5.44 | 86.22 ± 5.57 | 1097 (32.3) | 51 (4.6) |  |
| Cluster 2 | 96.06 ± 5.19 | 97.50 ± 5.41 | 1461 (43.0) | 102 (7.0) |  |
| Cluster 3 | 107.82 ± 6.40 | 109.12 ± 6.77 | 842 (24.8) | 79 (9.4) |  |
| **TyG-ABSI longitudinal change pattern** | | | | | <0.001 |
| Cluster 1 | 0.65 ± 0.04 | 0.65 ± 0.04 | 1069 (31.4) | 53 (5.0) |  |
| Cluster 2 | 0.71 ± 0.04 | 0.72 ± 0.04 | 1445 (42.5) | 92 (6.4) |  |
| Cluster 3 | 0.79 ± 0.05 | 0.79 ± 0.05 | 886 (26.1) | 87 (9.8) |  |
| **TyG-RFM longitudinal change pattern** | | | | | 0.102 |
| Cluster 1 | 193.15 ± 29.60 | 195.08 ± 32.46 | 1146 (33.7) | 69 (6.0) |  |
| Cluster 2 | 279.80 ± 30.66 | 285.62 ± 30.68 | 981 (28.9) | 61 (6.2) |  |
| Cluster 3 | 364.72 ± 32.73 | 371.93 ± 32.63 | 1273 (37.4) | 102 (8.0) |  |

**Table S7. Predictive performance of TyG-obesity indices for stroke by ROC analysis**

|  | AUC (95% CI) | P value | Sensitivity | Specificity | Youden | Best threshold |
| --- | --- | --- | --- | --- | --- | --- |
| **Baseline level** | | | | | | |
| TyG index | 0.567 (0.531 ~ 0.604) | reference | 0.828 | 0.306 | 0.134 | 8.259 |
| TyG-CVAI | 0.626 (0.590 ~ 0.663) | 0.0015 | 0.584 | 0.634 | 0.218 | 835.479 |
| TyG-BRI | 0.609 (0.573 ~ 0.645) | 0.0016 | 0.582 | 0.582 | 0.164 | 36.482 |
| TyG-CI | 0.607 (0.573 ~ 0.641) | 0.0067 | 0.301 | 0.862 | 0.163 | 10.358 |
| TyG-WWI | 0.604 (0.568 ~ 0.640) | 0.0110 | 0.487 | 0.667 | 0.154 | 99.545 |
| TyG-ABSI | 0.598 (0.561 ~ 0.634) | 0.0238 | 0.780 | 0.372 | 0.152 | 0.685 |
| TyG-RFM | 0.563 (0.524 ~ 0.601) | 0.8428 | 0.371 | 0.747 | 0.118 | 344.829 |
| **Cumulative exposure** | | | | | | |
| TyG Index | 0.553 (0.516 ~ 0.590) | reference | 0.642 | 0.450 | 0.092 | 33.850 |
| TyG-CVAI | 0.622 (0.585 ~ 0.659) | <0.0001 | 0.435 | 0.760 | 0.195 | 4265.407 |
| TyG-BRI | 0.600 (0.561 ~ 0.639) | 0.0293 | 0.552 | 0.604 | 0.156 | 152.554 |
| TyG-CI | 0.598 (0.562 ~ 0.635) | <0.0001 | 0.638 | 0.551 | 0.189 | 44.287 |
| TyG-WWI | 0.593 (0.555 ~0.630) | 0.0075 | 0.668 | 0.496 | 0.164 | 380.984 |
| TyG-ABSI | 0.590 (0.552 ~ 0.628) | 0.0067 | 0.634 | 0.539 | 0.173 | 2.854 |
| TyG-RFM | 0.558 (0.519 ~ 0.596) | 0.8811 | 0.409 | 0.700 | 0.109 | 1342.697 |

**Table S8. Incremental predictive value of baseline, cumulative, and longitudinal change in TyG-CVAI and TyG-BRI for stroke in CKM syndrome stages 0-3**

| Model | NRI (95%CI) | P value | IDI (95%CI) | P value |
| --- | --- | --- | --- | --- |
| Basic model | reference | reference | reference | reference |
| +Baseline TyG | 0.006 (-0.002~0.017) | 0.059 | 0.005 (-0.002~0.012) | 0.099 |
| +Cumulative TyG | 0.013 (-0.045~0.119) | 0.812 | -0.001 (-0.003~0.005) | 0.713 |
| +TyG longitudinal change | 0.085 (-0.025~0.196) | 0.128 | 0.003 (-0.002~0.014) | 0.291 |
| +Baseline TyG-CVAI | 0.141 (0.023~0.265) | 0.030 | 0.009 (0.001~0.026) | 0.020 |
| +Cumulative TyG-CVAI | 0.123 (0.029~0.255) | 0.020 | 0.007 (0.001~0.022) | 0.021 |
| +TyG-CVAI longitudinal change | 0.130 (0.058~0.238) | < 0.001 | 0.008 (0.002~0.025) | 0.020 |
| +Baseline TyG-BRI | 0.113 ( 0.035~0.208) | < 0.001 | 0.006 (0.001~0.021) | 0.048 |
| +Cumulative TyG-BRI | 0.080 (0.001~0.200) | 0.047 | 0.005 (0.001~0.019) | 0.047 |
| +TyG-BRI longitudinal change | 0.102 (0.011~0.198) | 0.020 | 0.006 (0.001~0.021) | 0.045 |

The basic model included age, sex, smoking, drinking, SBP, DBP, HDL-C, LDL-C, uric acid, hemoglobin, eGFR, hypertension, diabetes, chronic kidney disease, lung disease.

NRI, net reclassification improvement; Ref, reference; IDI, integrated discrimination improvement; CI, confidence interval; TyG, triglyceride-glucose index; TyG-CVAI, triglyceride glucose-Chinese visceral adiposity index; TyG-BRI, triglyceride glucose-body roundness index.

**Figure S3. Decision curve analysis for comparing TyG‑CVAI and basic model in stroke prediction in CKM syndrome stages 0-3**


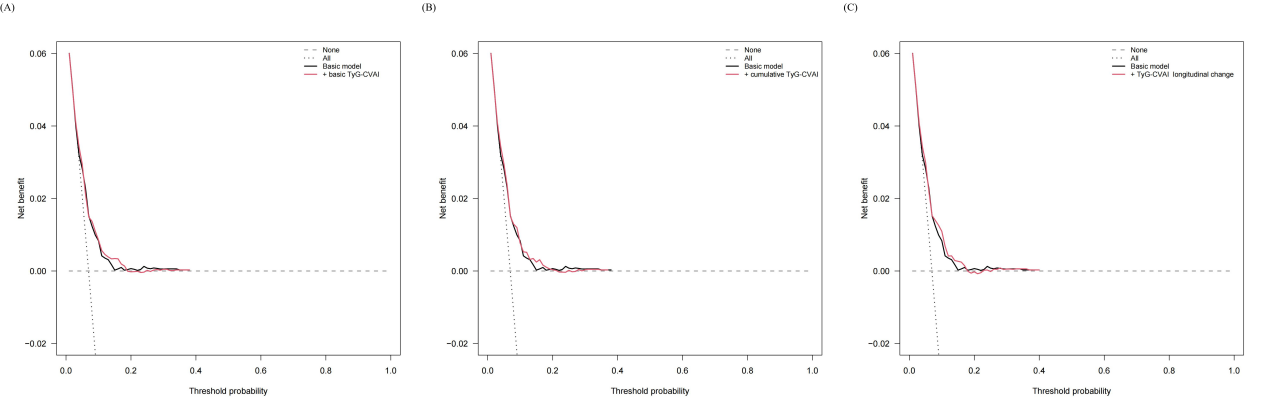


1. baseline TyG-CVAI, (B) cumulative TyG-CVAI, (C) TyG-CVAI longitudinal change. The y-axis represents net benefits, calculated by subtracting the relative harm (false positives) from the benefits (true positives). The x-axis calculates the threshold probability. The basic model included age, sex, smoking, drinking, SBP, DBP, HDL-C, LDL-C, uric acid, hemoglobin, eGFR, hypertension, diabetes, chronic kidney disease, lung disease.

.

**Figure S4. Decision curve analysis for comparing TyG‑BRI and basic model in stroke prediction in CKM syndrome stages 0-3**


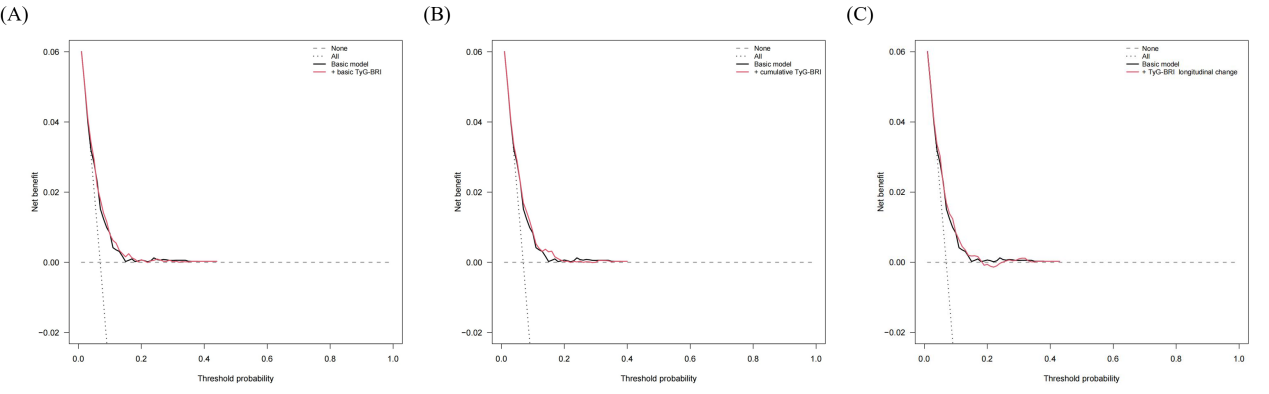


1. baseline TyG-BRI, (B) cumulative TyG-BRI, (C) TyG-BRI longitudinal change. The y-axis represents net benefits, calculated by subtracting the relative harm (false positives) from the benefits (true positives). The x-axis calculates the threshold probability. The basic model included age, sex, smoking, drinking, SBP, DBP, HDL-C, LDL-C, uric acid, hemoglobin, eGFR, hypertension, diabetes, chronic kidney disease, lung disease.

**Figure S5. Path diagram of the mediation analysis of ePWV**

**
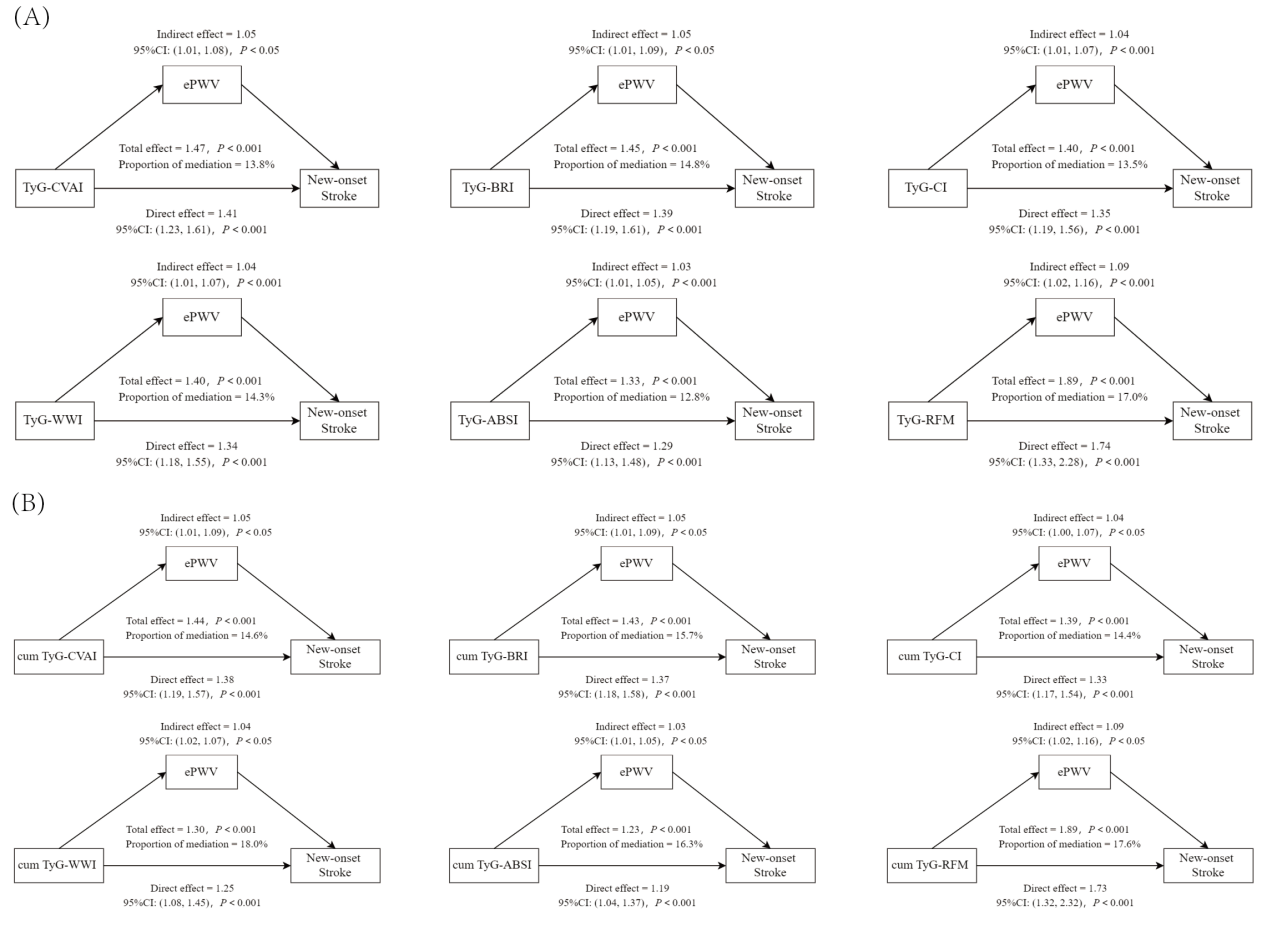
**

(A) Baseline TyG-obesity indices. (B) Cumulative TyG-obesity indices.

**Table S9. Association between baseline TyG-obesity indices and stroke in CKM syndrome stages 0-3: Excluding participants with stroke at wave 4 (n=3,278)**

| Variables | Event (%) | Model I | | Model II | | Model III | |
| --- | --- | --- | --- | --- | --- | --- | --- |
|  |  | HR (95% CI) | P value | HR (95% CI) | P value | HR (95% CI) | P value |
| Baseline TyG-CVAI |  |  |  |  |  |  |  |
| Tertile 1 (2.77-624.89) | 19 (1.7) | reference |  | reference |  | reference |  |
| Tertile 2 (625.42-934.27) | 34 (3.1) | 1.72 (0.98~3.01) |  | 1.69 (0.96~2.97) |  | 1.42 (0.80~2.52) |  |
| Tertile 3 (934.57-1895.51) | 57 (5.2) | 2.95 (1.76~4.97) | <0.001＊ | 2.82 (1.67~4.76) | <0.001＊ | 2.07 (1.17~3.64) | 0.012＊ |
| BaselineTyG-BRI |  |  |  |  |  |  |  |
| Tertile 1 (11.27-29.44) | 21 (1.9) | reference |  | reference |  | reference |  |
| Tertile 2 (29.45-39.80) | 32 (2.9) | 1.48 (0.85~2.57) |  | 1.63 (0.94~2.84) |  | 1.37 (0.78~2.40) |  |
| Tertile 3 (39.81-72.55) | 57 (5.2) | 2.57 (1.56~4.24) | <0.001＊ | 2.95 (1.75~4.97) | <0.001＊ | 2.12 (1.2~3.73) | 0.010＊ |
| Baseline TyG-CI |  |  |  |  |  |  |  |
| Tertile 1 (8.27-10.48) | 19 (1.7) | reference |  | reference |  | reference |  |
| Tertile 2 (10.49-11.45) | 41 (3.8) | 2.16 (1.25~3.72) |  | 2.12 (1.23~3.67) |  | 1.91 (1.10~3.33) |  |
| Tertile 3 (11.46-14.40) | 50 (4.6) | 2.73 (1.61~4.64) | <0.001＊ | 2.69 (1.57~4.61) | <0.001＊ | 1.97 (1.13~3.45) | 0.017＊ |
| Baseline TyG-WWI |  |  |  |  |  |  |  |
| Tertile 1 (71.27-90.73) | 22 (2.0) | reference |  | reference |  | reference |  |
| Tertile 2 (90.74-99.67) | 34 (3.1) | 1.55 (0.91~2.65) |  | 1.63 (0.95~2.80) |  | 1.40 (0.81~2.42) |  |
| Tertile 3 (99.68-128.52) | 54 (4.9) | 2.47 (1.51~4.06) | <0.001＊ | 2.67 (1.58~4.51) | <0.001＊ | 1.90 (1.10~3.29) | 0.022＊ |
| Baseline TyG-ABSI |  |  |  |  |  |  |  |
| Tertile 1 (0.53-0.68) | 23 (2.1) | reference |  | reference |  | reference |  |
| Tertile 2 (0.69-0.73) | 40 (3.7) | 1.80 (1.08~3.01) |  | 1.73 (1.03~2.90) |  | 1.51 (0.90~2.55) |  |
| Tertile 3 (0.74-0.93) | 47 (4.3) | 2.18 (1.32~3.59) | 0.002＊ | 2.06 (1.24~3.44) | 0.006＊ | 1.50 (0.89~2.55) | 0.130＊ |
| Baseline TyG-RFM |  |  |  |  |  |  |  |
| Tertile 1 (102.92-235.06) | 30 (2.7) | reference |  | reference |  | reference |  |
| Tertile 2 (235.11-328.33) | 36 (3.3) | 1.11 (0.68~1.80) |  | 1.97 (1.16~3.37) |  | 1.58 (0.89~2.79) |  |
| Tertile 3 (345.80-456.64) | 44 (4.0) | 1.34 (0.85~2.14) | 0.211＊ | 1.15 (0.93~1.45) | 0.170＊ | 1.07 (0.96~1.20) | 0.104＊ |
| Per SD increase |  |  |  |  |  |  |  |
| Baseline TyG-CVAI | 110 (3.4) | 1.62 (1.35~1.94) | <0.001 | 1.56 (1.30~1.88) | <0.001 | 1.39 (1.14~1.71) | 0.002 |
| Baseline TyG-BRI | 110 (3.4) | 1.42 (1.19~1.69) | <0.001 | 1.52 (1.26~1.83) | <0.001 | 1.30 (1.06~1.61) | 0.014 |
| Baseline TyG-CI | 110 (3.4) | 1.44 (1.20~1.73) | <0.001 | 1.43 (1.19~1.72) | <0.001 | 1.23 (1.01~1.51) | 0.042 |
| Baseline TyG-WWI | 110 (3.4) | 1.38 (1.15~1.65) | <0.001 | 1.42 (1.16~1.72) | 0.001 | 1.20 (0.97~1.49) | 0.088 |
| Baseline TyG-ABSI | 110 (3.4) | 1.36 (1.14~1.63) | 0.001 | 1.33 (1.10~1.60) | 0.003 | 1.17 (0.96~1.43) | 0.122 |
| Baseline TyG-RFM | 110 (3.4) | 1.16 (0.96~1.41) | 0.126 | 2.09 (1.47~2.96) | <0.001 | 1.56 (1.06~2.30) | 0.026 |

Model I unadjusted. Model II adjusted for age and sex. Model III adjusted for age, sex, smoking, drinking, SBP, DBP, marital status, hukou status, education, HDL-C, LDL-C, uric acid, hemoglobin, eGFR, hypertension, diabetes, chronic kidney disease, lung disease, antihypertensive agents, antidiabetic agents and antihyperlipidemic agents.＊P for trend.

**Table S10. Association between cumulative exposure and longitudinal change pattern in TyG-obesity indices and stroke in CKM syndrome stages 0-3: Excluding participants with stroke at wave 4 (n=3,278)**

| Variables | Event (%) | Model I | | Model II | | Model III | |
| --- | --- | --- | --- | --- | --- | --- | --- |
|  |  | HR (95% CI) | P value | HR (95% CI) | P value | HR (95% CI) | P value |
| Cumulative TyG-CVAI |  |  |  |  |  |  |  |
| Tertile 1 (87.65-2671.40) | 20 (1.8) | reference |  | reference |  | reference |  |
| Tertile 2 (2671.88-3907.92) | 33 (3.0) | 1.60 (0.92~2.79) |  | 1.65 (0.94~2.89) |  | 1.41 (0.80~2.50) |  |
| Tertile 3 (3908.50-7602.28) | 57 (5.2) | 2.70 (1.62~4.50) | <0.001＊ | 2.64 (1.58~4.42) | <0.001＊ | 1.97 (1.13~3.44) | 0.016＊ |
| Cumulative TyG-BRI |  |  |  |  |  |  |  |
| Tertile 1 (43.70-119.93) | 27 (2.5) | reference |  | reference |  | reference |  |
| Tertile 2 (119.93-161.86) | 25 (2.3) | 0.88 (0.51~1.52) |  | 0.99 (0.57~1.72) |  | 0.80 (0.45~1.41) |  |
| Tertile 3 (161.87-292.28) | 58 (5.3) | 1.93 (1.22~3.05) | 0.005＊ | 2.25 (1.38~3.25) | 0.001＊ | 1.65 (1.05~2.52) | 0.027＊ |
| Cumulative TyG-CI |  |  |  |  |  |  |  |
| Tertile 1 (32.65-42.18) | 24 (2.2) | reference |  | reference |  | reference |  |
| Tertile 2 (42.19-45.91) | 36 (3.3) | 1.32 (0.79~2.21) |  | 1.33 (0.79~2.24) |  | 1.13 (0.67~1.92) |  |
| Tertile 3 (45.92-56.55) | 50 (4.6) | 1.85 (1.13~3.00) | 0.014＊ | 1.86 (1.13~3.06) | 0.015＊ | 1.34 (0.79~2.26) | 0.276＊ |
| Cumulative TyG-WWI |  |  |  |  |  |  |  |
| Tertile 1 (282.51-364.81) | 24 (2.2) | reference |  | reference |  | reference |  |
| Tertile 2 (364.82-398.38) | 35 (3.2) | 1.33 (0.79~2.24) |  | 1.43 (0.84~2.42) |  | 1.18 (0.69~2.02) |  |
| Tertile 3 (398.39-505.17) | 51 (4.7) | 1.89 (1.16~3.06) | 0.010＊ | 2.05 (1.22~3.45) | 0.007＊ | 1.49 (0.86~2.57) | 0.155＊ |
| Cumulative TyG-ABSI |  |  |  |  |  |  |  |
| Tertile 1 (2.17-2.72) | 29 (2.7) | reference |  | reference |  | reference |  |
| Tertile 2 (2.73-2.94) | 33 (3.0) | 0.99 (0.60~1.64) |  | 0.98 (0.59~1.62) |  | 0.86 (0.51~1.43) |  |
| Tertile 3 (2.95-3.61) | 48 (4.4) | 1.46 (0.92~2.32) | 0.107＊ | 1.39 (0.87~2.24) | 0.172＊ | 1.01 (0.62~1.66) | 0.954＊ |
| Cumulative TyG-RFM |  |  |  |  |  |  |  |
| Tertile 1 (32.65-42.18) | 35 (3.2) | reference |  | reference |  | reference |  |
| Tertile 2 (42.19-45.91) | 28 (2.6) | 0.71 (0.43~1.17) |  | 1.27 (0.73~2.21) |  | 1.00 (0.55~1.81) |  |
| Tertile 3 (45.92-56.55) | 47 (4.3) | 1.17 (0.76~1.82) | 0.471＊ | 1.13 (0.81~1.29) | 0.344＊ | 1.05 (0.89~1.09) | 0.686＊ |
| Per SD increase |  |  |  |  |  |  |  |
| Cumulative TyG-CVAI | 110 (3.4) | 1.55 (1.29~1.86) | <0.001 | 1.51 (1.26~1.80) | <0.001 | 1.34 (1.09~1.65) | 0.005 |
| Cumulative TyG-BRI | 110 (3.4) | 1.39 (1.16~1.65) | <0.001 | 1.49 (1.24~1.80) | <0.001 | 1.30 (1.05~1.60) | 0.015 |
| Cumulative TyG-CI | 110 (3.4) | 1.29 (1.07~1.55) | 0.008 | 1.28 (1.06~1.55) | 0.010 | 1.10 (0.90~1.35) | 0.360 |
| Cumulative TyG-WWI | 110 (3.4) | 1.24 (1.03~1.49) | 0.024 | 1.27 (1.04~1.55) | 0.021 | 1.07 (0.86~1.33) | 0.546 |
| Cumulative TyG-ABSI | 110 (3.4) | 1.21 (1.00~1.45) | 0.046 | 1.18 (0.97~1.43) | 0.092 | 1.03 (0.84~1.26) | 0.798 |
| CumulativeTyG-RFM | 110 (3.4) | 1.11 (0.91~1.34) | 0.300 | 1.87 (1.31~2.67) | 0.001 | 1.39 (0.94~2.06) | 0.102 |
| TyG-CVAI longitudinal change | |  |  |  |  |  |  |
| Cluster 1 | 20 (1.8) | reference |  | reference |  | reference |  |
| Cluster 2 | 43 (3.2) | 1.75 (1.03~2.97) | 0.039 | 1.79 (1.05~3.07) | 0.032 | 1.57 (0.91~2.71) | 0.108 |
| Cluster 3 | 47 (6.0) | 3.34 (1.98~5.64) | <0.001 | 3.19 (1.88~5.42) | <0.001 | 2.35 (1.32~4.19) | 0.004 |
| TyG-BRI longitudinal change |  |  |  |  |  |  |  |
| Cluster 1 | 29 (2.3) | reference |  | reference |  | reference |  |
| Cluster 2 | 43 (3.2) | 1.32 (0.83~2.12) | 0.245 | 1.5 (0.93~2.43) | 0.098 | 1.22 (0.74~2.00) | 0.441 |
| Cluster 3 | 38 (5.4) | 2.15 (1.33~3.49) | 0.002 | 2.56 (1.53~4.30) | <0.001 | 1.84 (1.04~3.07) | 0.015 |
| TyG-CI longitudinal change |  |  |  |  |  |  |  |
| Cluster 1 | 22 (2.0) | reference |  | reference |  | reference |  |
| Cluster 2 | 48 (3.4) | 1.61 (0.97~2.67) | 0.065 | 1.66 (1.00~2.76) | 0.051 | 1.39 (0.83~2.33) | 0.213 |
| Cluster 3 | 40 (5.0) | 2.34 (1.39~3.93) | 0.001 | 2.39 (1.40~4.08) | 0.001 | 1.69 (0.96~2.97) | 0.071 |
| TyG-WWI longitudinal change | |  |  |  |  |  |  |
| Cluster 1 | 24 (2.2) | reference |  | reference |  | reference |  |
| Cluster 2 | 49 (3.5) | 1.51 (0.93~2.47) | 0.096 | 1.62 (0.99~2.67) | 0.057 | 1.36 (0.82~2.26) | 0.236 |
| Cluster 3 | 37 (4.6) | 2.00 (1.2~3.34) | 0.008 | 2.17 (1.25~3.77) | 0.006 | 1.47 (0.82~2.64) | 0.197 |
| TyG-ABSI longitudinal change | |  |  |  |  |  |  |
| Cluster 1 | 28 (2.7) | reference |  | reference |  | reference |  |
| Cluster 2 | 42 (3.0) | 1.09 (0.68~1.76) | 0.717 | 1.08 (0.66~1.75) | 0.761 | 0.90 (0.55~1.47) | 0.680 |
| Cluster 3 | 40 (4.8) | 1.75 (1.08~2.84) | 0.023 | 1.68 (1.01~2.78) | 0.044 | 1.18 (0.69~2.00) | 0.545 |
| TyG-RFM longitudinal change | |  |  |  |  |  |  |
| Cluster 1 | 35 (3.1) | reference |  | reference |  | reference |  |
| Cluster 2 | 28 (3.0) | 0.84 (0.51~1.38) | 0.482 | 1.38 (0.79~2.41) | 0.254 | 1.07 (0.59~1.94) | 0.812 |
| Cluster 3 | 47 (3.9) | 1.10 (0.71~1.71) | 0.663 | 1.03 (1.01~1.12) | 0.014 | 1.01 (0.72~1.09) | 0.204 |

Model I unadjusted. Model II adjusted for age and sex. Model III adjusted for age, sex, smoking, drinking, SBP, DBP, marital status, hukou status, education, HDL-C, LDL-C, uric acid, hemoglobin, eGFR, hypertension, diabetes, chronic kidney disease, lung disease, antihypertensive agents, antidiabetic agents and antihyperlipidemic agents.＊P for trend.

**Figure S6. Restricted cubic spline curve for the association between TyG-obesity indices and new-onset stroke : Excluding participants with new-onset stroke at wave 4 (n=3,278)**

**

**

Models were adjusted for age, sex, smoking, drinking, SBP, DBP, marital status, hukou status, education, HDL-C, LDL-C, uric acid, hemoglobin, eGFR, hypertension, diabetes, chronic kidney disease, lung disease, antihypertensive agents, antidiabetic agents and antihyperlipidemic agents.

**Figure S7. Subgroup analyses of associations between baseline TyG-CVAI/BRI and stroke**

**
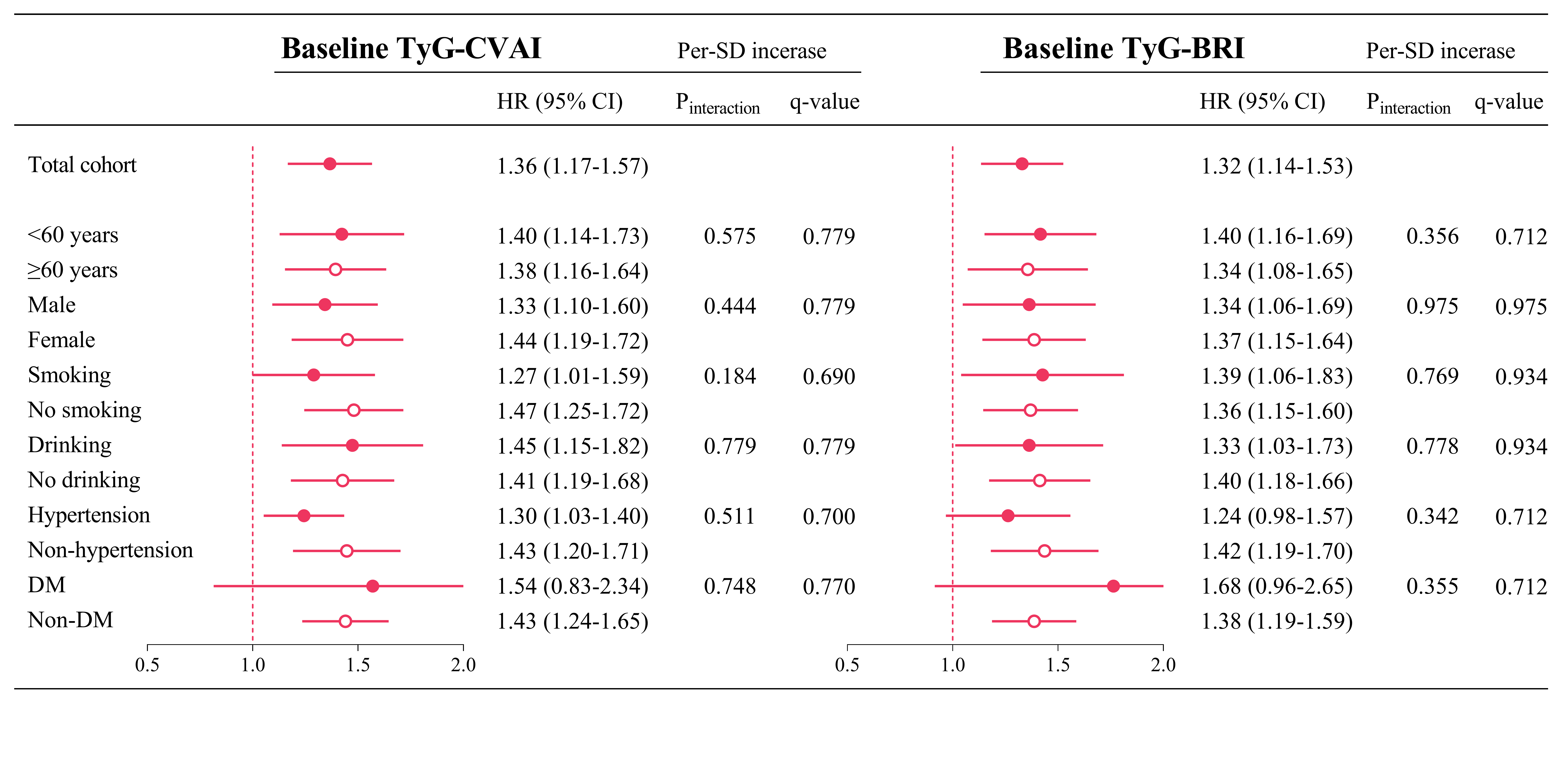
**

New-onset stroke in different strata as functions of 1-SD increase in baseline TyG-CVAI/BRI. Models were adjusted for age, sex, smoking, drinking, SBP, DBP, marital status, hukou status, education, HDL-C, LDL-C, uric acid, hemoglobin, eGFR, hypertension, diabetes, chronic kidney disease, lung disease, antihypertensive agents, antidiabetic agents and antihyperlipidemic agents. Not adjust for stratification variables. The Benjamini-Hochberg false discovery rate (FDR) procedure was applied to control for multiplicity in subgroup and interaction tests, with the q-values being reported.

**Figure S8. Subgroup analyses of associations between cumulative TyG-CVAI/BRI and stroke**

**
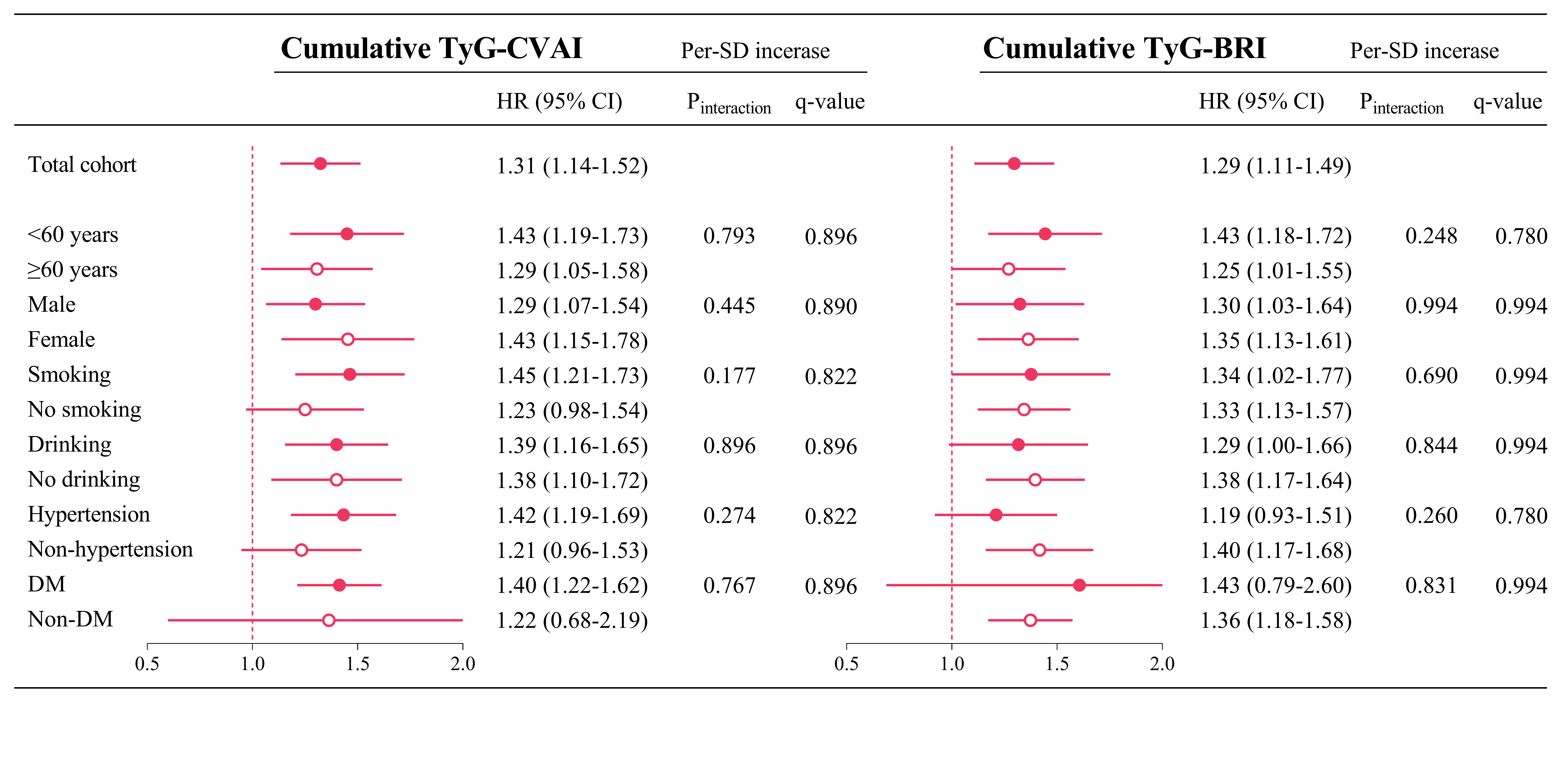
**

New-onset stroke in different strata as functions of 1-SD increase in cumulative TyG-CVAI/BRI. Models were adjusted for age, sex, smoking, drinking, SBP, DBP, marital status, hukou status, education, HDL-C, LDL-C, uric acid, hemoglobin, eGFR, hypertension, diabetes, chronic kidney disease, lung disease, antihypertensive agents, antidiabetic agents and antihyperlipidemic agents. Not adjust for stratification variables. The Benjamini-Hochberg false discovery rate (FDR) procedure was applied to control for multiplicity in subgroup and interaction tests, with the q-values being reported.

**Table S11. Subgroup analyses of associations between longitudinal change pattern of TyG-CVAI/BRI and stroke**

| Subgroup | | **TyG-CVAI** | | P_int_ | q‑  value | **TyG-BRI** | | P_int_ | q‑  value |
| --- | --- | --- | --- | --- | --- | --- | --- | --- | --- |
|  |  | Longitudinal change | HR (95% CI) |  |  | Longitudinal change | HR (95% CI) |  |  |
| Age (years) | <60 | Cluster 1 | reference | 0.691 | 0.711 | Cluster 1 | reference | 0.481 | 0.848 |
|  |  | Cluster 2 | 1.37 (0.81, 2.33) |  |  | Cluster 2 | 1.75 (1.07, 2.86) |  |  |
|  |  | Cluster 3 | 2.31 (1.34, 3.99) |  |  | Cluster 3 | 2.48 (1.43, 4.32) |  |  |
|  | ≥60 | Cluster 1 | reference |  |  | Cluster 1 | reference |  |  |
|  |  | Cluster 2 | 1.60 (0.94, 2.71) |  |  | Cluster 2 | 1.13 (0.69, 1.87) |  |  |
|  |  | Cluster 3 | 2.06 (1.16, 3.68) |  |  | Cluster 3 | 1.68 (0.95, 2.96) |  |  |
| Gender | Male | Cluster 1 | reference | 0.731 | 0.731 | Cluster 1 | reference | 0.512 | 0.788 |
|  |  | Cluster 2 | 1.49 (0.90, 2.44) |  |  | Cluster 2 | 1.18 (0.75, 1.85) |  |  |
|  |  | Cluster 3 | 1.92 (1.13, 3.28) |  |  | Cluster 3 | 2.02 (1.14, 3.56) |  |  |
|  | Female | Cluster 1 | reference |  |  | Cluster 1 | reference |  |  |
|  |  | Cluster 2 | 1.53 (0.86, 2.73) |  |  | Cluster 2 | 1.75 (0.96, 3.17) |  |  |
|  |  | Cluster 3 | 2.50 (1.35, 4.64) |  |  | Cluster 3 | 2.30 (1.24, 4.24) |  |  |
| Smoking | Yes | Cluster 1 | reference | 0.515 | 0.731 | Cluster 1 | reference | 0.645 | 0.848 |
|  |  | Cluster 2 | 1.66 (0.92, 2.97) |  |  | Cluster 2 | 1.29 (0.74, 2.27) |  |  |
|  |  | Cluster 3 | 1.82 (0.94, 3.55) |  |  | Cluster 3 | 2.39 (1.19, 4.81) |  |  |
|  | No | Cluster 1 | reference |  |  | Cluster 1 | reference |  |  |
|  |  | Cluster 2 | 1.48 (0.91, 2.41) |  |  | Cluster 2 | 1.54 (0.97, 2.44) |  |  |
|  |  | Cluster 3 | 2.43 (1.46, 4.06) |  |  | Cluster 3 | 2.00 (1.22, 3.29) |  |  |
| Drinking | Yes | Cluster 1 | reference | 0.191 | 0.690 | Cluster 1 | reference | 0.707 | 0.848 |
|  |  | Cluster 2 | 2.12 (1.15, 3.90) |  |  | Cluster 2 | 1.19 (0.69, 2.04) |  |  |
|  |  | Cluster 3 | 2.22 (1.13, 4.39) |  |  | Cluster 3 | 1.77 (0.89, 3.52) |  |  |
|  | No | Cluster 1 | reference |  |  | Cluster 1 | reference |  |  |
|  |  | Cluster 2 | 1.30 (0.81, 2.07) |  |  | Cluster 2 | 1.58 (0.99, 2.52) |  |  |
|  |  | Cluster 3 | 2.35 (1.44, 3.82) |  |  | Cluster 3 | 2.33 (1.41, 3.85) |  |  |
| Hypertension | Yes | Cluster 1 | reference | 0.507 | 0.710 | Cluster 1 | reference | 0.218 | 0.744 |
|  |  | Cluster 2 | 1.25 (0.66, 2.36) |  |  | Cluster 2 | 1.01 (0.59, 1.74) |  |  |
|  |  | Cluster 3 | 2.16 (1.17, 3.97) |  |  | Cluster 3 | 1.75 (1.00, 3.06) |  |  |
|  | No | Cluster 1 | reference |  |  | Cluster 1 | reference |  |  |
|  |  | Cluster 2 | 1.77 (1.12, 2.80) |  |  | Cluster 2 | 1.80 (1.14, 2.83) |  |  |
|  |  | Cluster 3 | 2.18 (1.26, 3.77) |  |  | Cluster 3 | 2.12 (1.19, 3.77) |  |  |
| Diabetes | Yes | Cluster 1 | reference | 0.325 | 0.722 | Cluster 1 | reference | 0.872 | 0.872 |
|  |  | Cluster 2 | 2.41 (0.65, 4.94) |  |  | Cluster 2 | 1.24 (0.41, 3.77) |  |  |
|  |  | Cluster 3 | 2.86 (0.76, 5.74) |  |  | Cluster 3 | 1.65 (0.53, 5.11) |  |  |
|  | No | Cluster 1 | reference |  |  | Cluster 1 | reference |  |  |
|  |  | Cluster 2 | 1.50 (1.02, 2.22) |  |  | Cluster 2 | 1.47 (1.02, 2.13) |  |  |
|  |  | Cluster 3 | 2.49 (1.65, 3.77) |  |  | Cluster 3 | 2.28 (1.50, 3.48) |  |  |

Models were adjusted for age, gender, smoking, drinking, SBP, DBP, marital status, hukou status, education, HDL-C, LDL-C, uric acid, hemoglobin, eGFR, hypertension, diabetes, chronic kidney disease, lung disease, antihypertensive agents, antidiabetic agents and antihyperlipidemic agents. Not adjust for stratification variables.

**Table S12. Associations of baseline, cumulative, and longitudinal change in TyG-CVAI with stroke in CKM syndrome stages 0-3: A Fine‑Gray competing risks analysis**

| Variable | Total | Event (%) | Model I | | Model II | | Model III | |
| --- | --- | --- | --- | --- | --- | --- | --- | --- |
|  |  |  | sHR (95%CI) | P value | sHR (95%CI) | P value | sHR (95%CI) | P value |
| Baseline TyG-CVAI | | | | | | | | |
| per SD | 3400 | 232 (6.8) | 1.51 (1.34~1.71) | <0.001 | 1.47 (1.30~1.66) | <0.001 | 1.51 (1.34~1.71) | <0.001 |
| Tertile1 | 1133 | 45 (4.0) | reference |  | reference |  | reference |  |
| Tertile2 | 1133 | 77 (6.8) | 1.72 (1.19~2.47) | 0.004 | 1.66 (1.15~2.39) | 0.007 | 1.49 (1.03~2.16) | 0.035 |
| Tertile3 | 1134 | 110 (9.7) | 2.47 (1.75~3.49) | <0.001 | 2.31 (1.64~3.27) | <0.001 | 1.79 (1.24~2.59) | 0.002 |
| Trend.test | 3400 | 232 (6.8) | 1.55 (1.32~1.82) | <0.001 | 1.50 (1.28~1.76) | <0.001 | 1.32 (1.11~1.57) | 0.002 |
| Cumulative TyG-CVAI | | | | | | | | |
| per SD | 3400 | 232 (6.8) | 1.48 (1.31~1.67) | <0.001 | 1.44 (1.27~1.62) | <0.001 | 1.31 (1.14~1.50) | <0.001 |
| Tertile1 | 1133 | 42 (3.7) | reference |  | reference |  | reference |  |
| Tertile2 | 1133 | 79 (7.0) | 1.89 (1.31~2.75) | 0.001 | 1.88 (1.29~2.75) | 0.001 | 1.71 (1.16~2.50) | 0.006 |
| Tertile3 | 1134 | 111 (9.8) | 2.65 (1.86~3.77) | <0.001 | 2.52 (1.77~3.58) | <0.001 | 2.00 (1.37~2.92) | <0.001 |
| Trend.test | 3400 | 232 (6.8) | 1.59 (1.35~1.86) | <0.001 | 1.54 (1.32~1.81) | <0.001 | 1.37 (1.15~1.63) | <0.001 |
| TyG-CVAI longitudinal change | | | | | | | | |
| Cluster1 | 1160 | 44 (3.8) | reference |  | reference |  | reference |  |
| Cluster2 | 1411 | 92 (6.5) | 1.74 (1.22~2.48) | 0.002 | 1.72 (1.20~2.47) | 0.003 | 1.57 (1.09~2.27) | 0.016 |
| Cluster3 | 829 | 96 (11.6) | 3.12 (2.19~4.44) | <0.001 | 2.92 (2.05~4.17) | <0.001 | 2.30 (1.56~3.39) | <0.001 |
| Trend.test | 3400 | 232 (6.8) | 1.77 (1.49~2.10) | <0.001 | 1.71 (1.44~2.03) | <0.001 | 1.51 (1.25~1.82) | <0.001 |

Model I unadjusted. Model II adjusted for age and sex. Model III adjusted for age, sex, smoking, drinking, SBP, DBP, marital status, hukou status, education, HDL-C, LDL-C, uric acid, hemoglobin, eGFR, hypertension, diabetes, chronic kidney disease, lung disease, antihypertensive agents, antidiabetic agents and antihyperlipidemic agents. CKM syndrome, cardiovascular kidney-metabolic syndrome; TyG-CVAI, triglyceride glucose-Chinese visceral adiposity index; sHR, subdistribution hazard ratio; CI, confidence interval.

**Table S13. Associations of baseline, cumulative, and longitudinal change of TyG-BRI with stroke in CKM syndrome stages 0–3: A Fine‑Gray competing risks analysis**

| Variable | Total | Event (%) | Model I | | Model II | | Model III | |
| --- | --- | --- | --- | --- | --- | --- | --- | --- |
|  |  |  | sHR (95%CI) | P value | sHR (95%CI) | P value | sHR (95%CI) | P value |
| Baseline TyG-BRI | | | | | | | | |
| per SD | 3400 | 232 (6.8) | 1.42 (1.26~1.60) | <0.001 | 1.46 (1.28~1.66) | <0.001 | 1.42 (1.26~1.60) | <0.001 |
| Tertile1 | 1133 | 45 (4.0) | reference |  | reference |  | reference |  |
| Tertile2 | 1133 | 77 (6.8) | 1.49 (1.05~2.14) | 0.027 | 1.58 (1.10~2.26) | 0.013 | 1.40 (0.98~2.01) | 0.065 |
| Tertile3 | 1134 | 110 (9.7) | 2.17 (1.56~3.03) | <0.001 | 2.28 (1.61~3.21) | <0.001 | 1.74 (1.20~2.53) | 0.004 |
| Trend.test | 3400 | 232 (6.8) | 1.47 (1.25~1.73) | <0.001 | 1.50 (1.27~1.77) | <0.001 | 1.31 (1.09~1.57) | 0.004 |
| Cumulative TyG-BRI | | | | | | | | |
| per SD | 3400 | 232 (6.8) | 1.39 (1.23~1.58) | <0.001 | 1.44 (1.26~1.64) | <0.001 | 1.29 (1.12~1.50) | 0.001 |
| Tertile1 | 1133 | 42 (3.7) | reference |  | reference |  | reference |  |
| Tertile2 | 1133 | 79 (7.0) | 1.30 (0.92~1.85) | 0.141 | 1.39 (0.97~1.99) | 0.074 | 1.22 (0.84~1.76) | 0.289 |
| Tertile3 | 1134 | 111 (9.8) | 1.97 (1.43~2.72) | <0.001 | 2.09 (1.49~2.93) | <0.001 | 1.58 (1.08~2.30) | 0.018 |
| Trend.test | 3400 | 232 (6.8) | 1.42 (1.20~1.66) | <0.001 | 1.45 (1.23~1.72) | <0.001 | 1.26 (1.05~1.52) | 0.015 |
| TyG-BRI longitudinal change | | | | | | | | |
| Cluster1 | 1275 | 44 (3.8) | reference |  | reference |  | reference |  |
| Cluster2 | 1381 | 92 (6.5) | 1.53 (1.10~2.12) | 0.011 | 1.67 (1.19~2.32) | 0.003 | 1.47 (1.04~2.08) | 0.030 |
| Cluster3 | 744 | 96 (11.6) | 2.45 (1.75~3.42) | <0.001 | 2.68 (1.88~3.82) | <0.001 | 2.03 (1.37~3.02) | <0.001 |
| Trend.test | 3400 | 232 (6.8) | 1.57 (1.33~1.85) | <0.001 | 1.63 (1.37~1.95) | <0.001 | 1.42 (1.17~1.73) | <0.001 |

Model I unadjusted. Model II adjusted for age and sex. Model III adjusted for age, sex, smoking, drinking, SBP, DBP, marital status, hukou status, education, HDL-C, LDL-C, uric acid, hemoglobin, eGFR, hypertension, diabetes, chronic kidney disease, lung disease, antihypertensive agents, antidiabetic agents and antihyperlipidemic agents. CKM syndrome, cardiovascular kidney-metabolic syndrome; TyG-CVAI, triglyceride glucose-Chinese visceral adiposity index; sHR, subdistribution hazard ratio; CI, confidence interval.

**Table S14. Comparison of baseline characteristics of included participants and those excluded due to missing TyG-related indices and CKM staging**

| Variables | Excluded population  (n = 14308) | Included population  (n = 3400) | P values | Standardized mean difference |
| --- | --- | --- | --- | --- |
| Age, years | 59.0 ± 10.6 | 58.3 ± 8.2 | < 0.001 | 0.070 (0.033~0.108) |
| Male, n (%) | 6915 (48.3) | 1564 (46.0) | 0.014 | 0.047 (0.009~0.084) |
| Smoking status, n (%) | 3836 (28.4) | 1035 (30.4) | 0.019 | 0.045 (0.007~0.082) |
| Drinking status, n (%) | 4615 (32.6) | 1152 (33.9) | 0.156 | 0.027 (-0.010~0.064) |
| Systolic blood pressure, mmHg | 130.3 ± 22.0 | 128.5 ± 20.2 | < 0.001 | 0.092 (0.053~0.131) |
| Diastolic blood pressure, mmHg | 75.7 ± 12.4 | 74.7 ± 11.9 | < 0.001 | 0.085 (0.046~0.124) |
| Married status, n (%) | 12356(86.6) | 3061(90.0) | < 0.001 | 0.108 (0.071~0.146) |
| Rural Hukou Status | 10789 (75.6) | 2905 (85.5) | < 0.001 | 0.252 (0.214~0.289) |
| High school education or above, n (%) | 1863 (13.1) | 305 (9.0) | < 0.001 | 0.130 (0.093~0.168) |
| Total cholesterol, mg/dL | 192.9 ± 39.6 | 193.1 ± 37.0 | 0.780 | 0.006 (-0.034~0.046) |
| HDL-C, mg/dL | 50.4 ± 15.6 | 51.9 ± 14.7 | < 0.001 | 0.100 (0.062~0.141) |
| LDL-C, mg/dL | 115.1 ± 35.5 | 118.2 ± 33.2 | < 0.001 | 0.091 (0.051~0.131) |
| HbA1c, % | 5.3 ± 0.9 | 5.2 ± 0.6 | < 0.001 | 0.100 (0.060~0.140) |
| Uric acid, mg/dL | 4.5 ± 1.3 | 4.3 ± 1.2 | < 0.001 | 0.168 (0.128~0.208) |
| Hemoglobin, g/dL | 14.4 ± 2.2 | 14.4 ± 2.2 | 0.136 | 0.041 (0.006~0.079) |
| eGFR, mL/min/1.73m2 | 96.2 ± 10.5 | 97.0 ± 12.6 | < 0.001 | 0.096 (0.046~0.146) |
| Hypertension, n (%) | 3761 (26.3) | 774 (22.7) | < 0.001 | 0.082 (0.045~0.119) |
| Diabetes mellitus, n (%) | 907 (6.3) | 155 (4.5) | < 0.001 | 0.078 (0.041~0.116) |
| Chronic kidney disease, n (%) | 1030 (7.2) | 183 (5.4) | < 0.001 | 0.075 (0.037~0.112) |
| Lung disease, n (%) | 1428 (10.0) | 276 (8.1) | < 0.001 | 0.065 (0.028~0.102) |
| Antihypertensive agents, n (%) | 2895 (20.6) | 519.0 (15.3) | < 0.001 | 0.139 (0.101~0.176) |
| Antidiabetic agents, n (%) | 622 (4.4) | 90.0 (2.6) | < 0.001 | 0.097 (0.059~0.134) |
| Antihyperlipidemic agents, n (%) | 727 (5.3) | 138.0 (4.1) | 0.004 | 0.057 (0.019~0.094) |

HDL-C, high-density lipoprotein cholesterol. LDL-C, low-density lipoprotein cholesterol. HbA1c, glycated haemoglobin.
