# Supplementary material for: Differential associations between novel triglyceride-glucose-obesity indices and incident stroke in individuals with early-stage cardiovascular-kidney-metabolic syndrome: a longitudinal cohort study
Source: Cardiovasc Diabetol. 2026 Jun 8;25:216. doi: 10.1186/s12933-026-03240-x (PMC13428451; doi:10.1186/s12933-026-03240-x)
Supplement: Supplementary file 2 — Supplementary Material 2. [file 12933_2026_3240_MOESM2_ESM.docx]

**Supplemental Material 2**

Table S1: Schoenfeld residual test of Cox regression model

| Variable | chi-square | df | p value |
| --- | --- | --- | --- |
| Baseline TyG-CVAI | 1.964 | 1 | 0.161 |
| Age | 0.097 | 1 | 0.755 |
| Gender | 0.162 | 1 | 0.687 |
| Marital status | 3.444 | 1 | 0.063 |
| Hukou status | 0.000 | 1 | 0.983 |
| Education | 3.819 | 1 | 0.053 |
| Smoking | 0.002 | 1 | 0.968 |
| Drinking | 0.193 | 1 | 0.660 |
| SBP | 3.457 | 1 | 0.063 |
| DBP | 1.139 | 1 | 0.286 |
| Hemoglobin | 1.927 | 1 | 0.165 |
| Uric acid | 0.007 | 1 | 0.934 |
| HDL-C | 0.554 | 1 | 0.457 |
| LDL-C | 2.894 | 1 | 0.089 |
| eGFR | 3.300 | 1 | 0.069 |
| Hypertension | 2.459 | 1 | 0.117 |
| Diabetes | 0.047 | 1 | 0.829 |
| CKD | 0.153 | 1 | 0.696 |
| Lung disease | 2.558 | 1 | 0.110 |
| Antidiabetic agents | 0.437 | 2 | 0.804 |
| Antihypertensive agents | 0.997 | 1 | 0.318 |
| Antihyperlipidemic agents | 3.533 | 1 | 0.060 |
| GLOBAL | 35.021 | 23 | 0.055 |

| Variable | chi-square | df | p value |
| --- | --- | --- | --- |
| Baseline TyG-BRI | 1.169 | 1 | 0.280 |
| Age | 0.087 | 1 | 0.768 |
| Gender | 0.177 | 1 | 0.674 |
| Smoking | 0.000 | 1 | 0.987 |
| Drinking | 0.212 | 1 | 0.645 |
| SBP | 3.486 | 1 | 0.062 |
| DBP | 1.198 | 1 | 0.274 |
| Marital status | 3.334 | 1 | 0.068 |
| Hukou status | 0.000 | 1 | 0.993 |
| Education | 3.798 | 1 | 0.051 |
| HDL-C | 0.590 | 1 | 0.442 |
| LDL-C | 2.902 | 1 | 0.088 |
| Uric acid | 0.005 | 1 | 0.942 |
| Hemoglobin | 1.801 | 1 | 0.180 |
| eGFR | 3.279 | 1 | 0.070 |
| Hypertension | 2.547 | 1 | 0.111 |
| Diabetes | 0.059 | 1 | 0.808 |
| CKD | 0.110 | 1 | 0.740 |
| Lung disease | 2.623 | 1 | 0.105 |
| Antidiabetic agents | 0.461 | 2 | 0.794 |
| Antihyperlipidemic agents | 3.622 | 2 | 0.164 |
| Antihypertensive agents | 0.977 | 1 | 0.323 |
| GLOBAL | 34.965 | 24 | 0.069 |

| Variable | chi-square | df | p value |
| --- | --- | --- | --- |
| Baseline TyG-CI | 0.408 | 1 | 0.523 |
| Age | 0.091 | 1 | 0.763 |
| Gender | 0.157 | 1 | 0.692 |
| Smoking | 0.003 | 1 | 0.959 |
| Drinking | 0.196 | 1 | 0.658 |
| SBP | 3.500 | 1 | 0.061 |
| DBP | 1.203 | 1 | 0.273 |
| Marital status | 3.389 | 1 | 0.066 |
| Hukou status | 0.000 | 1 | 0.993 |
| Education | 3.766 | 1 | 0.052 |
| HDL-C | 0.603 | 1 | 0.437 |
| LDL-C | 2.989 | 1 | 0.084 |
| Uric acid | 0.002 | 1 | 0.961 |
| Hemoglobin | 1.821 | 1 | 0.177 |
| eGFR | 3.403 | 1 | 0.065 |
| Hypertension | 2.525 | 1 | 0.112 |
| Diabetes | 0.07 | 1 | 0.791 |
| CKD | 0.113 | 1 | 0.737 |
| Lung disease | 2.555 | 1 | 0.11 |
| Antidiabetic agents | 0.476 | 2 | 0.788 |
| Antihyperlipidemic agents | 3.591 | 2 | 0.166 |
| Antihypertensive agents | 0.958 | 1 | 0.328 |
| GLOBAL | 34.214 | 24 | 0.081 |

| Variable | chi-square | df | p value |
| --- | --- | --- | --- |
| Baseline TyG-WWI | 0.153 | 1 | 0.696 |
| Age | 0.090 | 1 | 0.765 |
| Gender | 0.149 | 1 | 0.700 |
| Smoking | 0.003 | 1 | 0.957 |
| Drinking | 0.200 | 1 | 0.654 |
| SBP | 3.492 | 1 | 0.062 |
| DBP | 1.215 | 1 | 0.270 |
| Marital status | 3.384 | 1 | 0.066 |
| Hukou status | 0.000 | 1 | 0.998 |
| Education | 3.759 | 1 | 0.053 |
| HDL-C | 0.601 | 1 | 0.438 |
| LDL-C | 2.991 | 1 | 0.084 |
| Uric acid | 0.002 | 1 | 0.963 |
| Hemoglobin | 1.777 | 1 | 0.183 |
| eGFR | 3.395 | 1 | 0.065 |
| Hypertension | 2.538 | 1 | 0.111 |
| Diabetes | 0.066 | 1 | 0.797 |
| CKD | 0.111 | 1 | 0.739 |
| Lung disease | 2.550 | 1 | 0.110 |
| Antidiabetic agents | 0.474 | 2 | 0.789 |
| Antihyperlipidemic agents | 3.562 | 2 | 0.169 |
| Antihypertensive agents | 0.979 | 1 | 0.322 |
| GLOBAL | 34.063 | 24 | 0.084 |

| Variable | chi-square | df | p value |
| --- | --- | --- | --- |
| Baseline TyG-ABSI | 0.059 | 1 | 0.808 |
| Age | 0.091 | 1 | 0.763 |
| Gender | 0.144 | 1 | 0.704 |
| Smoking | 0.003 | 1 | 0.954 |
| Drinking | 0.195 | 1 | 0.659 |
| SBP | 3.499 | 1 | 0.061 |
| DBP | 1.209 | 1 | 0.272 |
| Marital status | 3.389 | 1 | 0.066 |
| Hukou status | 0.000 | 1 | 1.000 |
| Education | 3.767 | 1 | 0.052 |
| HDL-C | 0.604 | 1 | 0.437 |
| LDL-C | 3.038 | 1 | 0.081 |
| Uric acid | 0.002 | 1 | 0.965 |
| Hemoglobin | 1.765 | 1 | 0.184 |
| eGFR | 3.441 | 1 | 0.064 |
| Hypertension | 2.516 | 1 | 0.113 |
| Diabetes | 0.074 | 1 | 0.786 |
| CKD | 0.107 | 1 | 0.744 |
| Lung disease | 2.527 | 1 | 0.112 |
| Antidiabetic agents | 0.483 | 2 | 0.786 |
| Antihyperlipidemic agents | 3.552 | 2 | 0.169 |
| Antihypertensive agents | 0.975 | 1 | 0.323 |
| GLOBAL | 34.125 | 24 | 0.082 |

| Variable | chi-square | df | p value |
| --- | --- | --- | --- |
| Baseline TyG-RFM | 0.133 | 1 | 0.715 |
| Age | 0.102 | 1 | 0.749 |
| Gender | 0.151 | 1 | 0.698 |
| Marital status | 3.38 | 1 | 0.066 |
| Hukou status | 0.000 | 1 | 0.994 |
| Education | 3.776 | 1 | 0.052 |
| Smoking | 0.002 | 1 | 0.968 |
| Drinking | 0.19 | 1 | 0.663 |
| SBP | 3.475 | 1 | 0.062 |
| DBP | 1.172 | 1 | 0.279 |
| Hemoglobin | 1.838 | 1 | 0.175 |
| Uric acid | 0.004 | 1 | 0.949 |
| HDL-C | 0.561 | 1 | 0.454 |
| LDL-C | 2.888 | 1 | 0.089 |
| eGFR | 3.238 | 1 | 0.072 |
| Hypertension | 2.502 | 1 | 0.114 |
| Diabetes | 0.059 | 1 | 0.808 |
| CKD | 0.15 | 1 | 0.699 |
| Lung disease | 2.552 | 1 | 0.110 |
| Antidiabetic agents | 0.448 | 2 | 0.799 |
| Antihypertensive agents | 0.999 | 1 | 0.317 |
| Antihyperlipidemic agents | 3.529 | 1 | 0.060 |
| GLOBAL | 34.641 | 23 | 0.056 |

| Variable | chi-square | df | p value |
| --- | --- | --- | --- |
| Cumulative TyG-CVAI | 1.320 | 1 | 0.251 |
| Age | 0.096 | 1 | 0.757 |
| Gender | 0.153 | 1 | 0.695 |
| Smoking | 0.003 | 1 | 0.956 |
| Drinking | 0.208 | 1 | 0.648 |
| SBP | 3.509 | 1 | 0.061 |
| DBP | 1.173 | 1 | 0.279 |
| Marital status | 3.438 | 1 | 0.064 |
| Hukou status | 0.000 | 1 | 0.999 |
| Education | 3.828 | 1 | 0.053 |
| HDL-C | 0.561 | 1 | 0.454 |
| LDL-C | 2.889 | 1 | 0.089 |
| Uric acid | 0.010 | 1 | 0.922 |
| Hemoglobin | 1.850 | 1 | 0.174 |
| eGFR | 3.269 | 1 | 0.071 |
| Hypertension | 2.468 | 1 | 0.116 |
| Diabetes | 0.046 | 1 | 0.830 |
| CKD | 0.153 | 1 | 0.696 |
| Lung disease | 2.544 | 1 | 0.111 |
| Antidiabetic agents | 0.445 | 2 | 0.800 |
| Antihypertensive agents | 0.983 | 1 | 0.321 |
| Antihyperlipidemic agents | 3.556 | 1 | 0.059 |
| GLOBAL | 35.19 | 23 | 0.053 |

| Variable | chi-square | df | p value |
| --- | --- | --- | --- |
| Cumulative TyG-BRI | 0.862 | 1 | 0.353 |
| Age | 0.093 | 1 | 0.760 |
| Gender | 0.176 | 1 | 0.675 |
| Smoking | 0.001 | 1 | 0.972 |
| Drinking | 0.215 | 1 | 0.643 |
| SBP | 3.527 | 1 | 0.060 |
| DBP | 1.194 | 1 | 0.275 |
| Marital status | 3.456 | 1 | 0.063 |
| Hukou status | 0.000 | 1 | 0.989 |
| Education | 3.792 | 1 | 0.052 |
| HDL-C | 0.590 | 1 | 0.442 |
| LDL-C | 2.907 | 1 | 0.088 |
| Uric acid | 0.008 | 1 | 0.927 |
| Hemoglobin | 1.805 | 1 | 0.179 |
| eGFR | 3.251 | 1 | 0.071 |
| Hypertension | 2.552 | 1 | 0.110 |
| Diabetes | 0.057 | 1 | 0.812 |
| CKD | 0.109 | 1 | 0.741 |
| Lung disease | 2.612 | 1 | 0.106 |
| Antidiabetic agents | 0.455 | 2 | 0.797 |
| Antihyperlipidemic agents | 3.572 | 2 | 0.168 |
| Antihypertensive agents | 0.987 | 1 | 0.320 |
| GLOBAL | 35.229 | 24 | 0.065 |

| Variable | chi-square | df | p value |
| --- | --- | --- | --- |
| Cumulative TyG-CI | 0.200 | 1 | 0.654 |
| Age | 0.087 | 1 | 0.768 |
| Gender | 0.142 | 1 | 0.706 |
| Smoking | 0.003 | 1 | 0.956 |
| Drinking | 0.201 | 1 | 0.654 |
| SBP | 3.523 | 1 | 0.061 |
| DBP | 1.205 | 1 | 0.272 |
| Marital-status | 3.376 | 1 | 0.066 |
| Hukou status | 0.001 | 1 | 0.979 |
| Education | 3.783 | 1 | 0.052 |
| HDL-C | 0.597 | 1 | 0.440 |
| LDL-C | 2.977 | 1 | 0.084 |
| Uric acid | 0.005 | 1 | 0.941 |
| Hemoglobin | 1.723 | 1 | 0.189 |
| eGFR | 3.414 | 1 | 0.065 |
| Hypertension | 2.461 | 1 | 0.117 |
| Diabetes | 0.063 | 1 | 0.801 |
| CKD | 0.101 | 1 | 0.750 |
| Lung disease | 2.510 | 1 | 0.113 |
| Antidiabetic agents | 0.459 | 2 | 0.795 |
| Antihyperlipidemic agents | 3.496 | 2 | 0.174 |
| Antihypertensive agents | 1.000 | 1 | 0.317 |
| GLOBAL | 33.954 | 24 | 0.085 |

| Variable | chi-square | df | p value |
| --- | --- | --- | --- |
| Cumulative TyG-WWI | 0.040 | 1 | 0.841 |
| Age | 0.088 | 1 | 0.767 |
| Gender | 0.139 | 1 | 0.709 |
| Smoking | 0.003 | 1 | 0.956 |
| Drinking | 0.199 | 1 | 0.656 |
| SBP | 3.507 | 1 | 0.061 |
| DBP | 1.200 | 1 | 0.273 |
| Marital status | 3.375 | 1 | 0.066 |
| Hukou status | 0.001 | 1 | 0.980 |
| Education | 3.778 | 1 | 0.052 |
| HDL-C | 0.593 | 1 | 0.441 |
| LDL-C | 2.988 | 1 | 0.084 |
| Uric acid | 0.005 | 1 | 0.944 |
| Hemoglobin | 1.702 | 1 | 0.192 |
| eGFR | 3.422 | 1 | 0.064 |
| Hypertension | 2.464 | 1 | 0.116 |
| Diabetes | 0.063 | 1 | 0.801 |
| CKD | 0.099 | 1 | 0.753 |
| Lung disease | 2.504 | 1 | 0.114 |
| Antidiabetic agents | 0.458 | 2 | 0.795 |
| Antihyperlipidemic agents | 3.471 | 2 | 0.176 |
| Antihypertensive agents | 1.016 | 1 | 0.313 |
| GLOBAL | 33.864 | 24 | 0.087 |

| Variable | chi-square | df | p value |
| --- | --- | --- | --- |
| Cumulative TyG-ABSI | 0.001 | 1 | 0.979 |
| Age | 0.087 | 1 | 0.768 |
| Gender | 0.136 | 1 | 0.712 |
| Smoking | 0.003 | 1 | 0.956 |
| Drinking | 0.196 | 1 | 0.658 |
| SBP | 3.496 | 1 | 0.062 |
| DBP | 1.192 | 1 | 0.275 |
| Marital status | 3.363 | 1 | 0.067 |
| Hukou status | 0.001 | 1 | 0.981 |
| Education | 3.781 | 1 | 0.052 |
| HDL-C | 0.59 | 1 | 0.442 |
| LDL-C | 3.003 | 1 | 0.083 |
| Uric acid | 0.005 | 1 | 0.945 |
| Hemoglobin | 1.690 | 1 | 0.194 |
| eGFR | 3.446 | 1 | 0.063 |
| Hypertension | 2.448 | 1 | 0.118 |
| Diabetes | 0.065 | 1 | 0.799 |
| CKD | 0.095 | 1 | 0.757 |
| Lung disease | 2.492 | 1 | 0.114 |
| Antidiabetic agents | 0.458 | 2 | 0.795 |
| Antihyperlipidemic agents | 3.454 | 2 | 0.178 |
| Antihypertensive agents | 1.024 | 1 | 0.312 |
| GLOBAL | 33.887 | 24 | 0.087 |

| Variable | chi-square | df | p value |
| --- | --- | --- | --- |
| Cumulative TyG-RFM | 0.089 | 1 | 0.765 |
| Age | 0.102 | 1 | 0.750 |
| Gender | 0.148 | 1 | 0.701 |
| Marital status | 3.397 | 1 | 0.065 |
| Hukou status | 0.000 | 1 | 0.987 |
| Education | 3.778 | 1 | 0.052 |
| Smoking | 0.002 | 1 | 0.963 |
| Drinking | 0.190 | 1 | 0.663 |
| SBP | 3.507 | 1 | 0.061 |
| DBP | 1.188 | 1 | 0.276 |
| Hemoglobin | 1.768 | 1 | 0.184 |
| Uric acid | 0.006 | 1 | 0.939 |
| HDL-C | 0.562 | 1 | 0.453 |
| LDL-C | 2.933 | 1 | 0.087 |
| eGFR | 3.258 | 1 | 0.071 |
| Hypertension | 2.468 | 1 | 0.116 |
| Diabetes | 0.061 | 1 | 0.805 |
| CKD | 0.141 | 1 | 0.707 |
| Lung disease | 2.525 | 1 | 0.112 |
| Antidiabetic agents | 0.451 | 2 | 0.798 |
| Antihypertensive agents | 0.998 | 1 | 0.318 |
| Antihyperlipidemic agents | 3.508 | 1 | 0.061 |
| GLOBAL | 34.695 | 23 | 0.056 |

Table S2. Multicollinearity analysis of TyG-obesity indices and covariates in Model III

| Variable | GVIF | DF | GVIF^(1/(2*Df)) |
| --- | --- | --- | --- |
| SBP | 2.902 | 1 | 1.704 |
| DBP | 2.589 | 1 | 1.609 |
| Antidiabetic agents | 2.456 | 2 | 1.252 |
| Diabetes | 2.319 | 1 | 1.523 |
| Hypertension | 2.209 | 1 | 1.486 |
| Antihypertensive agents | 2.131 | 1 | 1.460 |
| Gender | 1.960 | 1 | 1.400 |
| **Baseline TyG-CVAI** | **1.810** | **1** | **1.345** |
| Age | 1.686 | 1 | 1.298 |
| HDL-C | 1.521 | 1 | 1.233 |
| Smoking | 1.520 | 1 | 1.233 |
| Drinking | 1.407 | 1 | 1.186 |
| eGFR | 1.338 | 1 | 1.157 |
| Uric acid | 1.255 | 1 | 1.120 |
| Antihyperlipidemic agents | 1.248 | 1 | 1.117 |
| Hemoglobin | 1.174 | 1 | 1.083 |
| Education | 1.116 | 1 | 1.056 |
| Marital status | 1.115 | 1 | 1.056 |
| Hukou status | 1.083 | 1 | 1.041 |
| LDL-C | 1.074 | 1 | 1.036 |
| CKD | 1.054 | 1 | 1.027 |
| Lung disease | 1.031 | 1 | 1.015 |

| Variable | GVIF | DF | GVIF^(1/(2*Df)) |
| --- | --- | --- | --- |
| SBP | 2.922 | 1 | 1.709 |
| DBP | 2.596 | 1 | 1.611 |
| Antidiabetic agents | 2.420 | 2 | 1.247 |
| Diabetes | 2.273 | 1 | 1.508 |
| Hypertension | 2.207 | 1 | 1.486 |
| Gender | 2.187 | 1 | 1.479 |
| Antihypertensive agents | 2.130 | 1 | 1.459 |
| Age | 1.660 | 1 | 1.288 |
| **Baseline TyG-BRI** | **1.612** | **1** | **1.270** |
| Smoking | 1.528 | 1 | 1.236 |
| Drinking | 1.397 | 1 | 1.182 |
| eGFR | 1.339 | 1 | 1.157 |
| Uric acid | 1.249 | 1 | 1.118 |
| Antihyperlipidemic agents | 1.234 | 1 | 1.111 |
| HDL-C | 1.224 | 1 | 1.106 |
| Hemoglobin | 1.167 | 1 | 1.080 |
| Marital status | 1.118 | 1 | 1.057 |
| Education | 1.115 | 1 | 1.056 |
| Hukou status | 1.074 | 1 | 1.036 |
| LDL-C | 1.069 | 1 | 1.034 |
| CKD | 1.056 | 1 | 1.028 |
| Lung disease | 1.030 | 1 | 1.015 |

| Variable | GVIF | DF | GVIF^(1/(2*Df)) |
| --- | --- | --- | --- |
| SBP | 2.927 | 1 | 1.711 |
| DBP | 2.597 | 1 | 1.611 |
| Antidiabetic agents | 2.515 | 2 | 1.259 |
| Diabetes | 2.355 | 1 | 1.535 |
| Hypertension | 2.211 | 1 | 1.487 |
| Antihypertensive agents | 2.131 | 1 | 1.460 |
| Gender | 2.071 | 1 | 1.439 |
| Age | 1.683 | 1 | 1.297 |
| **Baseline TyG-CI** | **1.618** | **1** | **1.272** |
| Smoking | 1.516 | 1 | 1.231 |
| Drinking | 1.405 | 1 | 1.185 |
| Uric acid | 1.377 | 1 | 1.174 |
| eGFR | 1.341 | 1 | 1.158 |
| HDL-C | 1.331 | 1 | 1.154 |
| Antihyperlipidemic agents | 1.245 | 1 | 1.116 |
| Hemoglobin | 1.165 | 1 | 1.079 |
| Education | 1.114 | 1 | 1.056 |
| Marital status | 1.111 | 1 | 1.054 |
| Hukou status | 1.068 | 1 | 1.033 |
| LDL-C | 1.062 | 1 | 1.030 |
| CKD | 1.057 | 1 | 1.028 |
| Lung disease | 1.032 | 1 | 1.016 |

| Variable | GVIF | DF | GVIF^(1/(2*Df)) |
| --- | --- | --- | --- |
| SBP | 2.934 | 1 | 1.713 |
| DBP | 2.600 | 1 | 1.612 |
| Antidiabetic agents | 2.504 | 2 | 1.258 |
| Diabetes | 2.344 | 1 | 1.531 |
| Gender | 2.267 | 1 | 1.506 |
| Hypertension | 2.208 | 1 | 1.486 |
| Antihypertensive agents | 2.135 | 1 | 1.461 |
| **Baseline TyG-WWI** | **1.764** | **1** | **1.328** |
| Age | 1.709 | 1 | 1.307 |
| Smoking | 1.514 | 1 | 1.231 |
| Drinking | 1.400 | 1 | 1.183 |
| Uric acid | 1.367 | 1 | 1.169 |
| eGFR | 1.343 | 1 | 1.159 |
| HDL-C | 1.299 | 1 | 1.140 |
| Antihyperlipidemic agents | 1.244 | 1 | 1.115 |
| Hemoglobin | 1.162 | 1 | 1.078 |
| Education | 1.115 | 1 | 1.056 |
| Marital status | 1.110 | 1 | 1.053 |
| Hukou status | 1.068 | 1 | 1.033 |
| LDL-C | 1.062 | 1 | 1.031 |
| CKD | 1.058 | 1 | 1.028 |
| Lung disease | 1.032 | 1 | 1.016 |

| Variable | GVIF | DF | GVIF^(1/(2*Df)) |
| --- | --- | --- | --- |
| SBP | 2.938 | 1 | 1.714 |
| DBP | 2.599 | 1 | 1.612 |
| Antidiabetic agents | 2.535 | 2 | 1.262 |
| Diabetes | 2.368 | 1 | 1.539 |
| Hypertension | 2.209 | 1 | 1.486 |
| Antihypertensive agents | 2.137 | 1 | 1.462 |
| Gender | 2.045 | 1 | 1.430 |
| Age | 1.707 | 1 | 1.307 |
| Smoking | 1.515 | 1 | 1.231 |
| **Baseline TyG-ABSI** | **1.493** | **1** | **1.222** |
| Drinking | 1.400 | 1 | 1.183 |
| Uric acid | 1.368 | 1 | 1.170 |
| eGFR | 1.341 | 1 | 1.158 |
| HDL-C | 1.272 | 1 | 1.128 |
| Antihyperlipidemic agents | 1.243 | 1 | 1.115 |
| Hemoglobin | 1.160 | 1 | 1.077 |
| Education | 1.114 | 1 | 1.056 |
| Marital status | 1.108 | 1 | 1.052 |
| Hukou status | 1.067 | 1 | 1.033 |
| CKD | 1.058 | 1 | 1.029 |
| LDL-C | 1.055 | 1 | 1.027 |
| Lung disease | 1.033 | 1 | 1.016 |

| Variable | GVIF | DF | GVIF^(1/(2*Df)) |
| --- | --- | --- | --- |
| Gender | 4.691 | 1 | 2.163 |
| **Baseline TyG-RFM** | **4.071** | **1** | **2.020** |
| SBP | 2.926 | 1 | 1.711 |
| DBP | 2.602 | 1 | 1.613 |
| Antidiabetic agents | 2.453 | 2 | 1.252 |
| Diabetes | 2.309 | 1 | 1.519 |
| Hypertension | 2.211 | 1 | 1.487 |
| Antihypertensive agents | 2.134 | 1 | 1.461 |
| Age | 1.653 | 1 | 1.286 |
| Smoking | 1.520 | 1 | 1.233 |
| Drinking | 1.399 | 1 | 1.183 |
| eGFR | 1.340 | 1 | 1.158 |
| HDL-C | 1.319 | 1 | 1.148 |
| Uric acid | 1.314 | 1 | 1.146 |
| Antihyperlipidemic agents | 1.244 | 1 | 1.115 |
| Hemoglobin | 1.166 | 1 | 1.080 |
| Marital status | 1.115 | 1 | 1.056 |
| Education | 1.115 | 1 | 1.056 |
| LDL-C | 1.076 | 1 | 1.037 |
| Hukou status | 1.072 | 1 | 1.035 |
| CKD | 1.056 | 1 | 1.028 |
| Lung disease | 1.030 | 1 | 1.015 |

| Variable | GVIF | DF | GVIF^(1/(2*Df)) |
| --- | --- | --- | --- |
| SBP | 2.910 | 1 | 1.706 |
| DBP | 2.596 | 1 | 1.611 |
| Antidiabetic agents | 2.453 | 2 | 1.252 |
| Diabetes | 2.315 | 1 | 1.521 |
| Hypertension | 2.205 | 1 | 1.485 |
| Antihypertensive agents | 2.132 | 1 | 1.460 |
| Gender | 1.969 | 1 | 1.403 |
| Age | 1.684 | 1 | 1.298 |
| **Cumulative TyG-CVAI** | **1.661** | **1** | **1.289** |
| Smoking | 1.515 | 1 | 1.231 |
| HDL-C | 1.421 | 1 | 1.192 |
| Drinking | 1.404 | 1 | 1.185 |
| eGFR | 1.337 | 1 | 1.156 |
| Uric acid | 1.244 | 1 | 1.115 |
| Antihyperlipidemic agents | 1.244 | 1 | 1.115 |
| Hemoglobin | 1.169 | 1 | 1.081 |
| Education | 1.117 | 1 | 1.057 |
| Marital status | 1.116 | 1 | 1.056 |
| Hukou status | 1.082 | 1 | 1.040 |
| LDL-C | 1.076 | 1 | 1.037 |
| CKD | 1.053 | 1 | 1.026 |
| Lung disease | 1.031 | 1 | 1.015 |

| Variable | GVIF | DF | GVIF^(1/(2*Df)) |
| --- | --- | --- | --- |
| SBP | 2.923 | 1 | 1.710 |
| DBP | 2.602 | 1 | 1.613 |
| Antidiabetic agents | 2.406 | 2 | 1.245 |
| Diabetes | 2.262 | 1 | 1.504 |
| Gender | 2.210 | 1 | 1.487 |
| Hypertension | 2.208 | 1 | 1.486 |
| Antihypertensive agents | 2.141 | 1 | 1.463 |
| Age | 1.656 | 1 | 1.287 |
| **Cumulative TyG-BRI** | **1.586** | **1** | **1.259** |
| Smoking | 1.520 | 1 | 1.233 |
| Drinking | 1.396 | 1 | 1.182 |
| eGFR | 1.335 | 1 | 1.155 |
| Antihyperlipidemic agents | 1.240 | 1 | 1.113 |
| Uric acid | 1.234 | 1 | 1.111 |
| HDL-C | 1.213 | 1 | 1.102 |
| Hemoglobin | 1.159 | 1 | 1.077 |
| Education | 1.116 | 1 | 1.056 |
| Marital status | 1.115 | 1 | 1.056 |
| Hukou status | 1.075 | 1 | 1.037 |
| LDL-C | 1.067 | 1 | 1.033 |
| CKD | 1.055 | 1 | 1.027 |
| Lung disease | 1.030 | 1 | 1.015 |

| Variable | GVIF | DF | GVIF^(1/(2*Df)) |
| --- | --- | --- | --- |
| SBP | 2.942 | 1 | 1.715 |
| DBP | 2.605 | 1 | 1.614 |
| Antidiabetic agents | 2.490 | 2 | 1.256 |
| Diabetes | 2.336 | 1 | 1.529 |
| Hypertension | 2.216 | 1 | 1.489 |
| Antihypertensive agents | 2.141 | 1 | 1.463 |
| Gender | 2.094 | 1 | 1.447 |
| Age | 1.673 | 1 | 1.293 |
| **Cumulative TyG-CI** | **1.593** | **1** | **1.262** |
| Smoking | 1.507 | 1 | 1.228 |
| Drinking | 1.402 | 1 | 1.184 |
| Uric acid | 1.336 | 1 | 1.156 |
| eGFR | 1.336 | 1 | 1.156 |
| HDL-C | 1.302 | 1 | 1.141 |
| Antihyperlipidemic agents | 1.248 | 1 | 1.117 |
| Hemoglobin | 1.159 | 1 | 1.077 |
| Education | 1.115 | 1 | 1.056 |
| Marital status | 1.107 | 1 | 1.052 |
| LDL-C | 1.073 | 1 | 1.036 |
| Hukou status | 1.069 | 1 | 1.034 |
| CKD | 1.058 | 1 | 1.029 |
| Lung disease | 1.031 | 1 | 1.015 |

| Variable | GVIF | DF | GVIF^(1/(2*Df)) |
| --- | --- | --- | --- |
| SBP | 2.944 | 1 | 1.716 |
| DBP | 2.606 | 1 | 1.614 |
| Antidiabetic agents | 2.491 | 2 | 1.256 |
| Diabetes | 2.337 | 1 | 1.529 |
| Gender | 2.331 | 1 | 1.527 |
| Hypertension | 2.213 | 1 | 1.487 |
| Antihypertensive agents | 2.142 | 1 | 1.464 |
| **Cumulative TyG-WWI** | **1.768** | **1** | **1.330** |
| Age | 1.701 | 1 | 1.304 |
| Smoking | 1.506 | 1 | 1.227 |
| Drinking | 1.396 | 1 | 1.182 |
| eGFR | 1.336 | 1 | 1.156 |
| Uric acid | 1.328 | 1 | 1.152 |
| HDL-C | 1.269 | 1 | 1.127 |
| Antihyperlipidemic agents | 1.247 | 1 | 1.116 |
| Hemoglobin | 1.158 | 1 | 1.076 |
| Education | 1.116 | 1 | 1.056 |
| Marital status | 1.106 | 1 | 1.052 |
| LDL-C | 1.071 | 1 | 1.035 |
| Hukou status | 1.068 | 1 | 1.034 |
| CKD | 1.058 | 1 | 1.028 |
| Lung disease | 1.030 | 1 | 1.015 |

| Variable | GVIF | DF | GVIF^(1/(2*Df)) |
| --- | --- | --- | --- |
| SBP | 2.944 | 1 | 1.716 |
| DBP | 2.603 | 1 | 1.613 |
| Antidiabetic agents | 2.503 | 2 | 1.258 |
| Diabetes | 2.347 | 1 | 1.532 |
| Hypertension | 2.213 | 1 | 1.488 |
| Antihypertensive agents | 2.142 | 1 | 1.464 |
| Gender | 2.081 | 1 | 1.443 |
| Age | 1.703 | 1 | 1.305 |
| Smoking | 1.507 | 1 | 1.228 |
| **Cumulative TyG-ABSI** | **1.501** | **1** | **1.225** |
| Drinking | 1.398 | 1 | 1.182 |
| eGFR | 1.336 | 1 | 1.156 |
| Uric acid | 1.335 | 1 | 1.155 |
| HDL-C | 1.257 | 1 | 1.121 |
| Antihyperlipidemic agents | 1.244 | 1 | 1.116 |
| Hemoglobin | 1.157 | 1 | 1.076 |
| Education | 1.115 | 1 | 1.056 |
| Marital status | 1.104 | 1 | 1.051 |
| Hukou status | 1.068 | 1 | 1.033 |
| LDL-C | 1.066 | 1 | 1.032 |
| CKD | 1.059 | 1 | 1.029 |
| Lung disease | 1.032 | 1 | 1.016 |

| Variable | GVIF | DF | GVIF^(1/(2*Df)) |
| --- | --- | --- | --- |
| Gender | 4.714 | 1 | 2.161 |
| **Cumulative TyG-RFM** | **4.001** | **1** | **2.000** |
| SBP | 2.937 | 1 | 1.714 |
| DBP | 2.607 | 1 | 1.615 |
| Antidiabetic agents | 2.458 | 2 | 1.252 |
| Diabetes | 2.311 | 1 | 1.520 |
| Hypertension | 2.214 | 1 | 1.488 |
| Antihypertensive agents | 2.141 | 1 | 1.463 |
| Age | 1.649 | 1 | 1.284 |
| Smoking | 1.513 | 1 | 1.230 |
| Drinking | 1.396 | 1 | 1.182 |
| eGFR | 1.336 | 1 | 1.156 |
| HDL-C | 1.285 | 1 | 1.133 |
| Uric acid | 1.283 | 1 | 1.133 |
| Antihyperlipidemic agents | 1.247 | 1 | 1.117 |
| Hemoglobin | 1.161 | 1 | 1.077 |
| Education | 1.116 | 1 | 1.056 |
| Marital status | 1.112 | 1 | 1.055 |
| LDL-C | 1.079 | 1 | 1.039 |
| Hukou status | 1.072 | 1 | 1.035 |
| CKD | 1.056 | 1 | 1.028 |
| Lung disease | 1.030 | 1 | 1.015 |
